# Measuring Energy Metabolism in the Mouse – Theoretical, Practical, and Analytical Considerations

**DOI:** 10.3389/fphys.2013.00034

**Published:** 2013-03-14

**Authors:** John R. Speakman

**Affiliations:** ^1^Key State Laboratory of Molecular Developmental Biology, Institute of Genetics and Developmental Biology, Chinese Academy of SciencesBeijing, China; ^2^Institute of Biological and Environmental Sciences, University of AberdeenAberdeen, Scotland, UK

**Keywords:** energy metabolism, indirect calorimetry, mouse models, energy balance, obesity, physical activity, basal metabolic rate, energy expenditure

## Abstract

The mouse is one of the most important model organisms for understanding human genetic function and disease. This includes characterization of the factors that influence energy expenditure and dysregulation of energy balance leading to obesity and its sequelae. Measuring energy metabolism in the mouse presents a challenge because the animals are small, and in this respect it presents similar challenges to measuring energy demands in many other species of small mammal. This paper considers some theoretical, practical, and analytical considerations to be considered when measuring energy expenditure in mice. Theoretically total daily energy expenditure is comprised of several different components: basal or resting expenditure, physical activity, thermoregulation, and the thermic effect of food. Energy expenditure in mice is normally measured using open flow indirect calorimetry apparatus. Two types of system are available – one of which involves a single small Spartan chamber linked to a single analyzer, which is ideal for measuring the individual components of energy demand. The other type of system involves a large chamber which mimics the home cage environment and is generally configured with several chambers/analyzer. These latter systems are ideal for measuring total daily energy expenditure but at present do not allow accurate decomposition of the total expenditure into its components. The greatest analytical challenge for mouse expenditure data is how to account for body size differences between individuals. This has been a matter of some discussion for at least 120 years. The statistically most appropriate approach is to use analysis of covariance with individual aspects of body composition as independent predictors.

## Overview

The mouse is probably the most important species as a model for the study of human diseases and disorders. Despite millions of years of evolutionary divergence the mouse has extremely close synteny of its genome with the human (Peltonen and McKusick, 2001), and physiologically, being a mammal and an endotherm, it shares many features of human metabolism not found in the other animal models such as ectothermic invertebrates like *Drosophila melanogaster* and *Caenorhabditis elegans*. The rat is also a mammalian endotherm that shares much of its genome and physiology with the human. What sets the mouse apart, however, is the technological capability to manipulate its genome to generate animals with global and tissue specific knock-out and transgenic models. This gives us phenomenal capabilities to explore the relationships between individual and multiple genes and their phenotypic consequences. Ascertaining the functions of the 30,000 or so genes in the human genome will be facilitated enormously by the study of the mouse in the coming decades.

Part of this effort will be to understand the impact that individual genes have on energy metabolism, and their consequences for disorders such as obesity (Speakman et al., 2008; Hall et al., 2012). This paper concerns theoretical and practical considerations for measuring the energy metabolism of the mouse. It also addresses the issue of how to analyze the resulting data and some of the pitfalls in this analysis. These considerations apply more generally to other small mammals in the same size range as mice (i.e., <100 g). I will therefore also draw on some examples in the literature of studies on such animals. Several other publications contain useful information on similar issues that are directly pertinent to the measurement of energy metabolism in the mouse and the reader may also wish to consult these, in particular the papers by Weir (1949), Kleiber (1961), Ferrannini (1988), Simonson and deFronzo (1990), Bursztein et al. (1989), Elia and Livesey (1992), Even et al. (1994), Arch et al. (2006), Lighton (2008), Tschoep et al. (2012), Even and Nadkarni (2012), Speakman et al. (2013).

## Theoretical Considerations

### Daily energy expenditure and its components

The total daily expenditure of energy (variously called TEE, TDEE, or DEE) can be partitioned into different components. These normally include the energy spent on basal metabolism, the thermic effect of food (the increase in energy expenditure following food intake which is also called the heat increment of feeding or the specific dynamic action), the energy spent on thermoregulation and the energy spent on physical activity. These components are often presented as a tower block shaded in different ways to reflect the different components and their relative sizes. However, a fundamental assumption being made in this type of diagram is that the components, as defined, are independent and additive. This may not be the case. Heat generated by activity or feeding, for example, may substitute for the costs of thermoregulation in some circumstances (Zerba and Walsberg, 1992; Bruinzeel and Piersma, 1998; Bech and Praesteng, 2004; Humphries and Careau, 2011; Virtue et al., 2012). Researchers may be interested in the impact of a given manipulation on the total daily energy expenditure and/or the components of expenditure. Ability to accurately measure the total daily expenditure or the different components depends on the type of equipment available.

### Indirect versus direct calorimetry for mouse measurements

**Figure 1:**
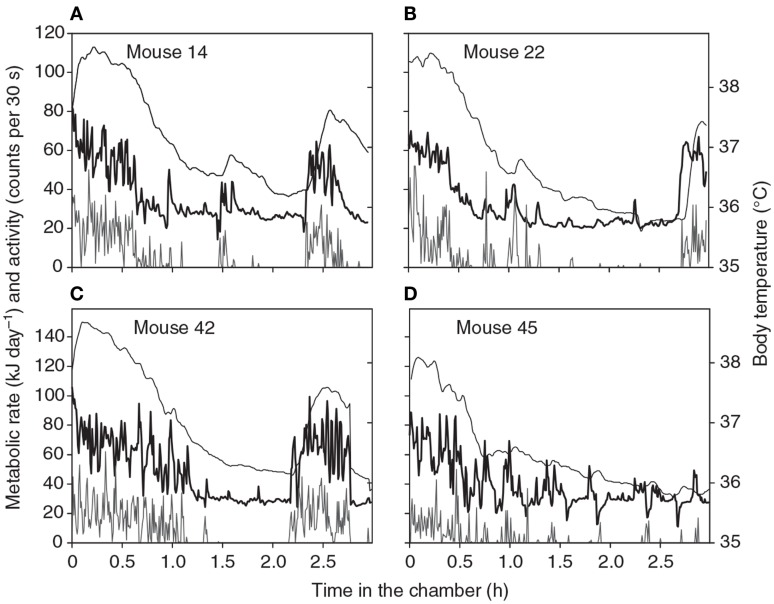
**Patterns of energy expenditure (thick black line: kJ/day) body temperature (thin black line: °C) and physical activity (gray line: “counts”/30 s) during four 3-h long respirometry measurements of four different individual mice**. Data are from a single chamber-single analyzer system with a small 1.25 L chamber. Measurements made every 30 s (from Duarte et al., 2010).

There are two fundamentally different ways to measure energy metabolism. The end product of all metabolic activity is either heat or work. Since work also ultimately appears as heat, one way is to measure the heat produced directly by the animal. This is called direct calorimetry. Direct calorimetry was popular in the first half of the last century but it fell out of favor because it is difficult to use, mostly because measuring small amounts of heat is technically challenging. Moreover, it makes a critical assumption that no heat is stored in the animals’ body during the measurement period. The error induced by this assumption can be quite large. Imagine a 30 g mouse is in a direct calorimeter and it is expending 0.35 W. If its body temperature was to rise by 1°C over the course of an hour in the chamber then it would have stored 125.5 J of heat (assuming the specific heat capacity of body tissue is about the same as that for water). This would be equivalent to 0.035 W (125.5/3,600), or 10% of the metabolic rate. So the actual measured heat production would be 10% too low. Equally if it cooled down by 1°C then the heat production estimate would be 10% higher than the actual metabolism, by virtue of the released body heat. Such changes in body temperature may routinely occur during measurements of energy expenditure (Figure 1).

The alternative is to not measure the heat directly but rather measure components of the metabolic process that generate the heat, and hence infer its production indirectly. This has become known as indirect calorimetry, or, because respiratory gases are used, respirometry.

The overall equation for the metabolism of glucose for example is

(1) $${\text{C}}_{6}{\text{H}}_{12}{\text{O}}_{6}+6({\text{H}}_{2}\text{O})+6({\text{O}}_{2})\to 12({\text{H}}_{2}\text{O})+6({\text{CO}}_{\text{2}})+2820\text{ kJ}$$

Hence we know from this equation that for every 6 mol of oxygen consumed or CO_2_ produced that the animal has used 2,820 kJ of energy. Fortunately measuring oxygen and CO_2_ gases can be performed with great accuracy: much better accuracy than for the small amounts of heat involved. Moreover, these compounds are not normally stored in the body to any great extent – unlike heat. The only downside of this approach is that animals do not always metabolize glucose, and when they change to burning other substrates the equation changes. However the equation changes in a systematic way depending on the substrate being used. This can be diagnosed from the measured ratio of oxygen consumption to CO_2_ production (called the respiratory exchange ratio: RER). The actual substrate oxidation at the tissue level is called the respiratory quotient (RQ). RQ is reflected in the RER but because of lags in the body they are not directly equivalent over short timescales. If we can work out how much nitrogen has been produced via the urine to calculate protein oxidation, then we can work out the other substrate oxidations from the RER (Weir, 1949), and very accurately calculate the energy expenditure. In fact, not correcting for differences in protein oxidation induces only a small error, unless protein oxidation exceeds 15% (Even and Nadkarni, 2012) and most people ignore this effect, using only the oxygen consumption combined with the estimated RQ from simultaneous measurements of CO_2_ production. The result is in theory accurate to within 1–2% of the true energy expenditure (Weir, 1949; Ferrannini, 1988), but see Walsberg and Hoffman (2005) for data showing that in practice discrepancies can be much higher. An argument has been made that assumptions underlying indirect calorimetry methodology remain untested in genetically modified mice and using both direct and indirect calorimetry in tandem may be a useful way forwards (Kaiyala and Ramsay, 2011).

The first systems to measure oxygen consumption of mice were closed systems (Davis and van Dyke, 1932, 1933). The animals were placed in a sealed chamber with a chemical that absorbs CO_2_ (generally calcium carbonate or soda lime) and the resultant consumption of oxygen altered the internal pressure which could be measured using a manometer. These systems, however, have two problems. First, it is only possible with this system to measure oxygen consumption. In theory it is feasible to measure the RQ by omitting the CO_2_ absorber and measuring volume changes, but this is very inaccurate as the volume changes are small and confounded by water vapor changes. Second, the mouse inside the chamber can perform lots of other behaviors as well as resting and it is difficult to separate out these effects. A method to try and eliminate the major source of this artifact was developed in the 1930s and involved simply waiting until the mouse settled down before fully sealing the chamber (Davis and van Dyke, 1932), but if it wakes up again afterward the measure is compromised.

Many measurements have been made using such systems and, despite being unable to accurately diagnose the substrate being oxidized, because of no RQ estimate, the data generated are quite good. The reason for this is that the error in converting from oxygen consumption to energy demands in the absence of a known RQ is relatively small. Nevertheless, the inability to adequately separate resting and non-resting behavior, and the construction of gas analyzers that could measure gas concentrations continuously, led eventually to the development of open flow respirometry systems, and these currently dominate the field. Very few people still use closed systems or direct calorimetry. In an open flow system the chamber is connected into a continuous flow of gas. Hence the “sealed” chamber has an incurrent and excurrent gas flow. Typically the gas will be drawn from an atmospheric source by a pump and then dried using silica gel. The gas flow will then be measured and regulated by a mass flow controller before entering the chamber where the animal is placed. The chamber will also have an outflow, downstream of which the gases will be dried again to remove any moisture introduced by the animal, and then passed into the gas analyzer where O_2_, and/or CO_2_ concentrations will be measured. Sophisticated systems may have a parallel stream that does not contain an animal and passes into a second channel of the analyzer to provide a constant reference point for atmospheric gas levels. These dual flow analyzer systems provide the most accurate estimates of oxygen consumption and CO_2_ production. However, if there is only a single analyzer channel then the background oxygen and CO_2_ levels are generally imputed from the start and end concentrations, by performing a “drift” correction. Generally when the measurement period is short (<3 to 4 h) then making a single start to end drift correction is adequate. However, if the measurement is longer it is often necessary to interrupt the measurement to obtain a background estimate. This is not as simple as it may first appear because the chamber where the animal resides must continue to be ventilated at exactly the same rate while the background is being measured.

This level of flow control only became technologically possible with the advent of mass flow controllers that allowed a regulated fixed flow of gas to enter the chamber independent of the pump supplying the gas. Whether the reference measurement is obtained directly, or imputed from single or multiple drift corrections, the measured or calculated difference in gas concentrations between the reference and the sample channels provides an estimate of the O_2_ and CO_2_ concentration changes produced by the animal in the chamber. The maths for calculating the oxygen consumption and CO_2_ production in such systems were worked out many years ago and summarized by Weir (1949), see also Even et al. (1994), Arch et al. (2006), Lighton (2008).

A point to note here is the importance of drawing the incurrent gas stream from outside the room where the measurements are being made, preferably a completely fresh airstream. This is because the background CO_2_ and O_2_ contents of the room atmosphere can be significantly impacted by the presence of the researcher or other staff in the room. This may compromise the assumption of linear drift. In addition it may not be obvious but the position of the pump has a bearing on the reliability of the system and influences exactly where the flow rate should be regulated and measured. As a general rule the flow rate should be regulated and measured immediately adjacent to the pump. If the pump is placed upstream of the chamber the system runs under slight positive pressure, and if it is placed downstream it is under slight negative pressure. This influences what happens if there is a slight leak in the chamber. In a system running under positive pressure (pump in incurrent stream) some gas will leak out of the chamber. This will be at the same concentration as that exiting down the excurrent tube to the analyzer, so will not influence the analyzer reading, but clearly if you pump xx ml into the chamber but <xx ml goes down the excurrent tube, if you monitor the flow in the excurrent tube rather than adjacent to the pump, in the incurrent tube, you will have an error in the flowrate equal to the magnitude of the leak. Similarly if the system runs under negative pressure (pump in excurrent flow) then if there is a slight leak in the chamber, atmospheric gases will be drawn in via the leak as well as via the incurrent tube. Again if you draw xx ml out of the chamber via the excurrent tube but <xx is coming in via the incurrent tube you will have an error in the flowrate the magnitude of the leak if you monitor and regulate the flow via the incurrent stream, rather than adjacent to the pump in the excurrent flow. The exact calculation is also dependent on whether the flow is measured upstream or downstream of the chamber (for details refer to Weir, 1949; Ferrannini, 1988; Even et al., 1994; Arch et al., 2006).

If both CO_2_ and O_2_ are measured then the resultant oxygen consumption can be converted into an energy expenditure measurement using the inferred substrate utilization (RQ) from the measured RER, assuming negligible protein oxidation has occurred. However, if only oxygen (or only CO_2_) is measured then it is necessary to assume an RQ value to derive the energy expenditure. Unless there is good reason to expect the animal is metabolizing exclusively fat, or a known diet with a given composition (food quotient) then generally the unknown RQ is assumed to be 0.8 or 0.85, as values between 0.7 (pure fat oxidation) and 1.0 (pure carbohydrate oxidation) minimize the error in the assumption. It should be noted that the potential error for converting oxygen consumption to energy expenditure is much smaller than the potential error converting CO_2_ production to energy expenditure, when the actual RQ is unknown Hence, if sufficient resources are available only to purchase either an O_2_ analyzer or a CO_2_ analyzer, one is better to buy the O_2_ analyzer. Moreover, if you read literature based only on CO_2_ estimates then it is good to be aware of the potential errors involved in the extrapolation to energy when RQ is unknown. This also applies to the doubly labeled water and labeled bicarbonate methods (below) which measure only CO_2_ production.

An issue to be considered here is if one has only an O_2_ analyzer is it better to also absorb the CO_2_ as well as the water vapor from the stream of gas exiting the chamber (Arch et al., 2006). The reasoning behind this is that the CO_2_ dilutes the oxygen concentration to some unknown extent and this can introduce an error into the estimated VO_2_. If you are interested in measuring oxygen consumption then to obtain the most accurate estimate it is best to absorb both the CO_2_ and the water in the excurrent stream. The equations to use with this type of configuration were established over a century ago by Haldane (1912) and are reiterated in detail in Weir (1949), Even et al. (1994), and Arch et al. (2006). Perhaps surprisingly if you are interested in energy expenditure rather than oxygen consumption then this configuration does not give the most accurate result (Koteja, 1996; Speakman, 2000). The reason is that there are actually two assumptions and errors being made in the whole process of going from oxygen concentration measurements to energy expenditure. The first assumption, if the CO_2_ is not absorbed, is the extent of the dilution due to the unknown amount of CO_2_ present. The error resulting from this assumption depends on what the actual RQ is relative to the RQ that is assumed. However, there is a second assumption when converting the oxygen consumption into energy expenditure, and that is what the oxycalorific equivalent of the consumed oxygen is. There is consequently also an error that depends on the difference between the assumed and the actual RQ. These two errors almost completely cancel each other out (Koteja, 1996). The net result is that if you absorb the CO_2_ you get a more accurate estimate of O_2_ consumption, but a worse estimate of the energy expenditure because you have removed one of the two errors that cancel each other out. So the message is clear. If your primary interest is energy and not oxygen then do not absorb the CO_2_ from the excurrent stream of the respirometry chamber.

### Single channel or multichannel systems

In a single channel system, a single chamber is positioned in a gas flow that goes into a single analyzer. There may or may not be a second channel used as a reference channel but the main distinguishing point of these systems is that the animal in question is measured for the entire time it is in the chamber. The key problem with such systems is that unless you have lots of them (which is expensive) then measuring multiple animals is a slow process. If for example one was interested in characterizing mouse basal energy demands for which a 3–4 h measurement is typical it would be difficult to get more than one measurement into a standard working day (if prior starvation time is taken into account – see below), making the normal throughput about five animals/week. The invention of mass flow controllers however meant that several chambers could be simultaneously ventilated at exactly the same rate. So by constructing a switching mechanism to divert the excurrent flows from different chambers in various directions it is possible to get the analyzer to sequentially measure a series of chambers. A typical configuration might include eight chambers, but ones with 16 and even 32 chambers are also available. The key point about these systems is that there is still only 1 analyzer and that analyzer cannot measure two chambers at the same time. So each mouse in the system is measured for only part of the time. Theoretically one might imagine in an eight chamber system each mouse is measured only 1/8th (12.5%) of the time, but in fact this is not the case because in a switching system it is necessary to have a period between each switch where the system purges the gas currently in the system from the previous animal. So for example if it takes 2 min to purge the system and the chamber flips between chambers every 3 min then the system will be purging for two-thirds of the time and measuring for only one-third. Each animal will then be measured for 1 min every cycle around the eight chambers which will take 8 min × 3 min to complete. Hence instead of being continuously monitored as in a single chamber-single analyzer system each animal is measured for just 1 min every 24 min (4.1% of the time). Clearly as the numbers of chambers increases this “measurement” becomes less and less representative. For a 32 chamber system on the same 3 min cycle each animal would be measured for 1 min every 96 min (1.04% of the time). If the time/chamber is increased before flipping to the next one in the sequence then the percent time spent purging is reduced. For example if the time/chamber was increased from 3 to 10 min then the system would be purging only 20% of the time. Hence the animal would be measured for 8 min. However, that 8 min would come around much less frequently. In an eight chamber system only once every 80 min. The animal is now measured 10% of the time instead of the 4.1% of the time on the 3 min cycle, but the measurement depends on how representative that continuous 8 min is of the whole 80 min. Most researchers have tended to go for more rapid sampling to get a more even spread of the measured minutes across the whole measurement time. Hence while it may appear that these multichannel systems are measuring 8, 16, or 32 animals, in reality they are often measuring nothing, because they are purging the system, and when they do measure something they still only measure one animal. It has been argued that these factors compromise the use of such systems for accurate determination of energy expenditure (Even and Nadkarni, 2012).

These two types of system are actually designed to do very different jobs. The single channel one chamber one analyzer system, generally using a very small chamber is ideally designed for making measurements of components of the energy balance such as Basal metabolic rate (BMR), or thermoregulatory costs, or the costs of physical activity. They are also ideally suited to measuring the acute impacts of treatments with drugs or with compounds believed to impact on energy metabolism (e.g., Hoggard et al., 2004; Valle et al., 2008). However these systems are unable to measure daily energy demands because the chamber is too small for the animal to live in for any protracted period. The multichannel systems where several chambers feed into a single analyzer are designed to make exactly this latter type of measurement. In this case the chamber volume is much larger so that it can contain a food hopper and water dispenser, space for a nest and also space to allow the animal to move around. The larger chamber with a slow washout and infrequent monitoring is poorly suited to measuring the detailed components of energy metabolism. However this system is ideal for measuring daily energy demands. The slow washout characteristics integrate the animals metabolism over time, this is compatible with the infrequent chamber monitoring. In recent years there have been attempts to decompose the measures from these multiple chamber systems into the components of metabolism (van Klinken et al., 2012). At present these methods are poorly advanced and the resultant accuracy cannot match frequently sampling single chamber-single analyzer systems (Even and Nadkarni, 2012). However, multichannel systems have recently entered the market that work on a one chamber one analyzer principal (the Promethion system from Sable systems is an example). In these systems each chamber IS monitored continuously, generally also with a continuous reference measurement. Such systems are superior to the standard switching systems based on a single analyzer monitoring multiple chambers and using this system it may be possible to get the best of both worlds – a good daily energy expenditure measurement with an accurate decomposition of the components.

### Correcting measurements to standard temperature and pressure dry and SI units

Gas volumes change with temperature and pressure. Hence when we calculate the oxygen consumption of an animal by measuring the flow volume (as opposed to mass) and multiply that by the concentration differences in the airflow, the result that we get is dependent on the ambient temperature at which the air flow rate is controlled and the barometric pressure at the time the measurement is made. This is not the case if the mass of the flow is determined. Since temperature and pressure may vary over the time course of a measurement it is also often necessary to measure these at the start and end of each measurement and to assume linear drifts in these parameters as well. Alternatively some machines have an ambient temperature and pressure compensation system fitted. This basically measures the ambient pressure and temperature continuously and then exerts a back pressure into the flow to simulate a constant pressure of 760 mmHg and a temperature of 0°C. If such a device is not fitted the correction to standard temperature and pressure for dry air must be performed. In both cases the resultant oxygen consumption should be referred to as VO_2_ STPD. The SI unit for volume is the liter. For mice respiratory gas consumption or production is normally expressed in units of ml/min, or L/h. If corrections for temperature and pressure are made automatically by the instrument then it is important for the user to ascertain that the Standard Temperature and Pressure values that are used by the software of the indirect calorimetry system to derive the flow are equal to the STP values used in the tables in literature that contain the coefficients of energy expenditure/volume of O_2_ or CO_2_. In more recent years to avoid any confusion about standard temperatures and pressures it has become common in the comparative physiology literature to express oxygen consumption or CO_2_ production in mols of oxygen or CO_2_/unit time.

The SI unit for energy is the joule. Although the use of calories is common this is not an SI unit. The SI unit for the rate of energy expenditure is the Watt. One Watt is equal to 1 J/s. Energy expenditures of mice measured over periods of minutes and hours should normally be quoted in Watts. However, because the time base of the Watt is the second this gives a poor idea of the level of expenditure over a whole day, which in many cases is the variable of interest. Hence daily energy expenditures should normally be quoted in kJ/day.

## Practical Issues

### BMR, RMR, and RMRt

Basal metabolic rate, occasionally called BEE (basal energy expenditure), was introduced early last century to standardize measurements of metabolism across different species. The basic requirement for a measure to qualify as basal is that the organism should be at rest, alert (i.e., should normally not be sleeping), post-absorptive (i.e., not digesting food), not growing or reproducing, at a temperature within the thermoneutral zone and measured during the quiescent phase of its diurnal cycle. Although not initially prescribed it is also generally assumed that this animal it at its normal body temperature (euthermic) and in the quiescent phase of its daily cycle (Aschoff and Phol, 1970). Effects of time of day on the metabolism of mice have been known since at least the 1940s (Fuhrman et al., 1946). To qualify as a measure of resting metabolic rate the only criterion is that the animal should be at rest and euthermic. Many measurements of metabolic rate in mice fall between these two limits. That is they meet the criterion of being at thermoneutral, but it is not entirely clear if the animals are post-absorptive or not. Speakman et al. (2004) suggested the term RMRt should be used for these measurements, which is additionally useful for measurements that are otherwise basal, but made on growing or reproducing animals.

A key issue in measuring BMR in mice is the time needed to starve a mouse to make sure it is post-absorptive. Initially it was assumed that for most animals it was necessary to starve them overnight (Kleiber, 1961). However, not feeding overnight is a major energetic challenge to most small mammals the size of a mouse and in response they enable many defense mechanisms to conserve energy. This includes suppressing metabolic rate and lowering the body temperature. This leads to a situation where the requirements for BMR start to become mutually incompatible. The longer an animal is starved the more likely it is to be post-absorptive, but the less likely it is to be euthermic (see also Gallivan, 1992; Speakman et al., 1993; McNab, 1997 for discussion of this trade-off in the measurement of cetacean and soricid metabolic rates). In consideration of these issues many recent measurements for mice and other small rodents have used much shorter periods of starvation prior to the measurement of BMR in the range of 4–5 h (Ksiazek et al., 2004; Sadowska et al., 2009; Zhao et al., in review).

Although chamber size does not enter into the calculation of metabolic rate it has a large impact on metabolism measures. This is because the chamber acts as a mixing box for the respiratory gases. The concentration of oxygen and CO_2_ exiting the chamber is therefore a reflection of the integrated pattern of the oxygen consumption and CO_2_ production of the animal over a period of time. The duration of this time depends on the chamber size, chamber design, and the flow rate. The lower this time is the more closely the excurrent gas flow reflects the instantaneous metabolism of the animal being measured. In theory if a pulse of CO_2_ was introduced into a chamber at time 0, and there was perfect mixing in the chamber then the concentration of CO_2_ in the excurrent flow would decline exponentially back toward the baseline. The half life of this exponential decline is a measure of the chamber washout characteristics. It is dependent on the chamber volume and the flow rate. Higher flow rates and smaller chambers lead to faster washout characteristics. By making the washout faster the measured oxygen consumption more closely reflects the instantaneous metabolic rate of the animal being measured. There are however several trade-offs to be considered. If the chamber is too small it may be restrictive and the animal may be stressed by the confinement and have an elevated metabolic rate (Pertwee and Tavendale, 1977). So chamber volume can only be reduced to a certain extent. The smallest chambers we have successfully used for measuring BMR in mice are cylindrical chambers with a diameter of 6 cm and a length of 10 cm giving a chamber volume of 283 ml. The washout time can also be reduced by increasing the flow rate. However as the flow rate is increased the difference in oxygen and CO_2_ contents between incurrent and excurrent gas streams gets smaller and the consequence is potentially reduced precision in the estimated difference. For example, if one was measuring a mouse with a resting metabolic rate of 0.6 ml/min using a flow rate of 1,000 ml/min, the difference in oxygen concentration between the incurrent and excurrent flows would be only 0.06%. Ideally the difference between incurrent and excurrent oxygen and CO_2_ concentrations should be maintained above 0.2%, and ideally in the range 0.2–0.8%. This sets a limit on improving washout by increasing flow rates. However chamber volume and chamber flow rate are not the only factors influencing washout rates.

By strategically locating the inflow and outflow of the chamber, mixing in the chamber can be maximized. However, chamber design may have a significant impact on washout characteristics. Square or oblong chambers may have dead spaces in the corners that retard mixing in addition adding any form of complexity inside the chamber may also create dead spaces that impede mixing of the gases inside the chamber. Ideally for BMR and RMR measurements it is best to have as simple a chamber as possible. We use cylindrical chambers measuring 8 cm in diameter and 25 cm long with a perforated floor in the bottom that keeps the mouse separated from any feces or urine it may produce. The volume is 1,257 ml. Using a flow rate of 300 ml/min the half life for the washout is about 2.5 min, and the difference in oxygen concentration between inflow and outflow for a mouse consuming 0.6 ml/min is about 0.5%. This gives a balance between minimizing washout time, not restricting the animal too much, and maintaining a large enough incurrent-excurrent concentration difference of the respiratory gases to get a precise estimate.

The duration of a BMR measurement needs to be long enough for the animal to completely settle down within the chamber. It is often suggested that animals should be familiarized with the chamber environment on a number of trial runs prior to the actual measurements but we have not found any evidence that there are systematic differences in BMR measures between the first, second, third, and fourth experiences in the chamber (Duarte et al., 2010) using mice that had no prior exposure to the environment. Hence this preconditioning does not seem necessary. Four typical patterns of metabolic rate and simultaneous physical activity and body temperature during a respirometry measurement in a chamber like that described above are shown in Figure 1 (from Duarte et al., 2010). These measurements show some common features of all measurements we have made on mice. Initially there is much physical activity in the chamber. This seems to be exploratory and does not decline with repeated measurements. During this period the body temperature is also elevated to between 38 and 38.5°C. This phase generally lasts for about 30 min to an hour. The mouse then settles down (normally curled up and stationary) and the metabolic rate gradually declines. The decline in metabolism reflects a slow decline in body temperature to a stable level between 35.8 and 36.5°C. Both metabolic rate and body temperature normally reach a stable minimum after about 2 h. The animal may wake up move around and then go back to sleep. These periods of elevated physical activity correspond to periods of increased metabolic rate and body temperature. These are particularly noticeable in the traces for animals 14, 22, and 42. Occasionally there are dips in the metabolic rate (see especially the trace for animal 45). Not all animals show these dips and they are not observed on all repeat measurements in the same individual.

Measurements of BMR are generally made as the lowest observed metabolic rate over a pre-defined measurement period (for example 5 min – see Discussion below over the choice of this interval). However, the experimenter has a choice how long to leave the animal in the chamber waiting for a low 5 min period to be observed. In theory the longer an animal is left the lower the measurement will be. This is because if actual metabolic rates follow a Gaussian distribution around some mean value and one draws samples at random from this distribution, then the more samples you take the more likely you are to get a lower one than the previous lowest you observed (Hayes et al., 1992). We have looked at a large number of metabolism measurements in both mice and bank voles and found that the lowest 5 min measurement declines as the measurement duration is increased, mostly because of the change in physical activity, but this decline is not significant after about 2.5 h in mice. We therefore make our BMR measurements over 3 h. In bank voles we use 4 h because the decline in the estimate remains significant until about 3.5 h. Longer measurements may in any case become an issue as the animals are deprived of water whilst in the chamber.

The rate at which samples are collected from the analyzer and averaged is also a variable under experimenter control. Modern Analog to Digital conversion cards make thousands of conversions every second, so even sampling at a rate of several measurements/second is feasible. However, if the chamber washout characteristics mean that the chamber half life is measured in minutes, these high frequency measurements are not independent of each other, and variation between them more likely reflect equipment noise than any biological phenomenon. With a washout time of about 2.5 min in our system we typically make time-averaged measurements over 10–30 s intervals. In switching systems where a single analyzer pays attention to several chambers sequentially making a large number of high frequency measurements over the short interval that the chamber is being measured does not compensate for the short time each chamber is actually measured, as these high frequency measures are pseudo replicates.

**Figure 2:**
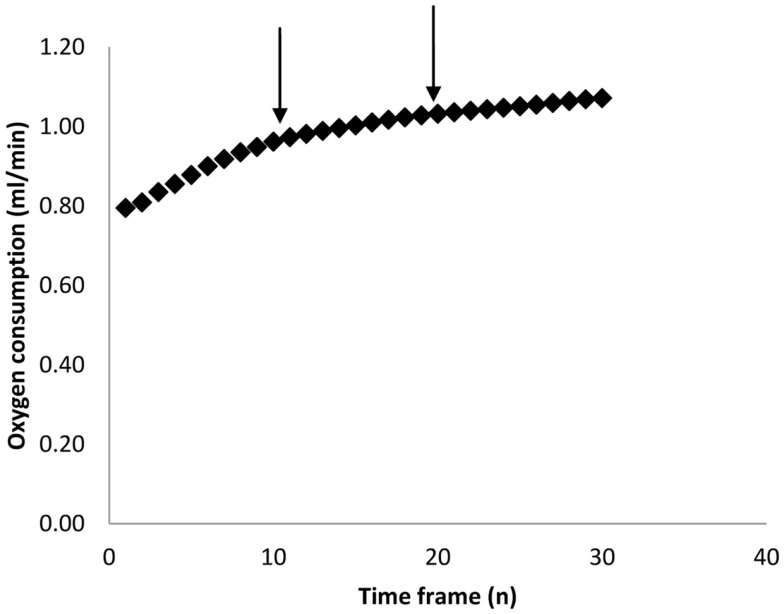
**Calculated minimum oxygen consumption over increasing time frames (*n* measurements)**. Each measurement lasted 30 s. Data are averaged across 43 individual MF1 mice involved in a study of weight loss on calorie restricted diets (Vaanholt et al., 2012). The arrows mark 5 and 10 min intervals which we have used in previous publications.

As mentioned above, most researchers characterize BMR as the lowest measured metabolic rate over some pre-defined period (e.g., 5 min). The estimate obtained however is theoretically (Hayes et al., 1992) and practically dependent on the duration of this interval. Figure 2 shows the empirical relationship between the estimated minimal metabolism and the duration over which the measurement is averaged for a typical sample of 50 metabolic rate measurements in mice (data from Vaanholt et al., 2012). There is a positive curvilinear relationship for all the measurements, but this seldom reaches an asymptote. This curve probably reflects the fact that at short intervals one is picking up stochastic variation in metabolism and/or noise in the equipment/system. These stochastic variations get smoothed out by extending the duration, but become more likely to then include brief periods of activity or small movements. Because there is no clear asymptote in this relationship the choice of the duration over which to average is arbitrary. In our system, with a chamber washout of 2.5 min, a sampling time of 5 min seems a reasonable compromise between avoiding stochastic variation on one side and including minor activities on the other. This choice however is specific to our system. In systems with larger chambers and longer washouts there will be an illusion of greater robustness to the choice of sample duration simply because these stochastic variations and minor activities are integrated by the chamber and not therefore detectable by the analyzer.

One feature evident from Figure 1 is the transient reductions in metabolic rate that are occasionally observed (see especially for animal 45). The cause of these reductions are unclear but they do not appear to be a machine artifact as they are never observed if the chambers are operated without an animal present and they consequently seem to be a real feature of metabolism. One possibility is that the animals simply stop breathing, and become apneic for a short period. Apnea is frequently observed in animals during torpor when the metabolic rate is extremely low (e.g., Hays et al., 1990; Thomas et al., 1990) and these data suggest it may also transiently occur during euthermy. Whatever the cause these dips in the record mean that any estimate of metabolism using an algorithm to detect the lowest × minutes of metabolism will always home in on the region surrounding such a phenomenon. This may consequently be a completely unrepresentative measure of the BMR. To avoid this problem we have started to also use an algorithm that detects the least variable *n* minutes of metabolism. This finds the most stable period of measurement, which is generally a period of low metabolism without any dip in it. If there is a discrepancy between the absolute lowest and the least variable we choose the least variable.

A key requirement for the measurement of BMR (or RMRt) is that the animal is at a thermoneutral temperature; that is it is measured within the thermoneutral zone (TNZ). Because evaporative water loss increases as one moves from the lower margin of the TNZ to the upper margin the most desirable temperature at which to measure BMR is around the lower critical temperature. This is particularly because in most metabolic chambers designed for BMR measurements the animals do not have access to water. In mice the *T*_lc_ has been estimated for various strains and is generally between 26 and 30°C (Hussein, 1991; Gordon, 1993; Speakman and Rossi, 1999; Selman et al., 2001; Golozoubova et al., 2004; Meyer et al., 2004; reviewed in Speakman and Keijer, 2013).

**Figure 3:**
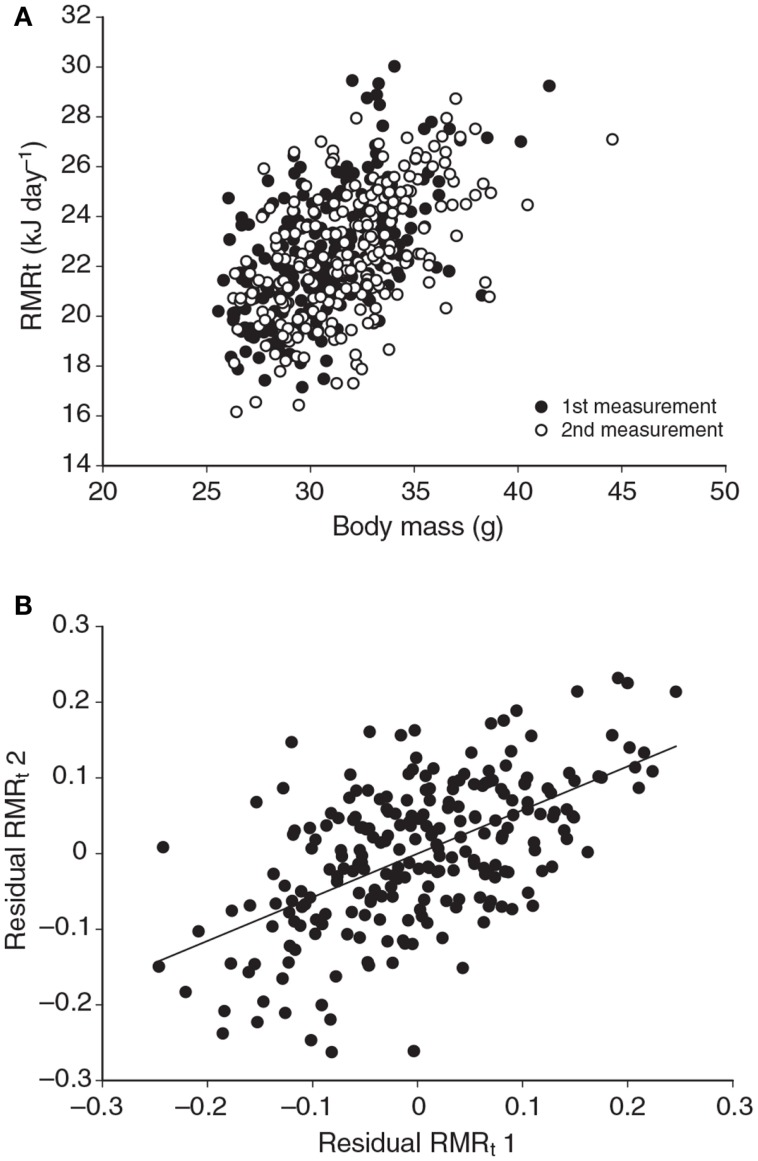
**(A)** Relationship between RMRt (resting metabolic rate measured at thermoneutral temperatures) and body mass for 323 mice measured on two occasions separated by about 15 days. There was a strong effect of body mass at both time points. **(B)** Residual measurements of RMRt, with the effect of body mass removed, measured at time 1 plotted against the same data at measurement time 2, for the same 323 mice. Residual RMRt was highly repeatable over this short timescale (data from Duarte et al., 2010).

Several studies have previously addressed the repeatability of BMR in small mammals including mice (Labocha et al., 2004; Russell and Chappell, 2007; Boratynski and Koteja, 2009; Duarte et al., 2010) and other energetic measurements such as DEE (Speakman et al., 1994; Fletcher et al., 2013). Repeatability of the measurement of BMR (or RMRt) in mice is important for two reasons. First repeatability sets a limit on heritability. Second, by knowing the repeatability of a measurement we can evaluate using power analysis the required sample size to detect a real difference in BMR following a given treatment in a repeated measures design (see below under analytical considerations). We have previously measured the repeatability of RMRt in the mouse and found that the measure is highly repeatable when measurements are separated by periods of about 15 days (Duarte et al., 2010). However, in part this repeatability is because there is a positive relationship between body mass and metabolic rate (Figure 3A) and animals tend to be consistent in their body masses. Nevertheless, if the residual metabolic rates are calculated (deviations from the fitted regression line between metabolism and body weight) these also show high repeatability when the interval between measurements is relatively short (Figure 3B). Over longer periods the repeatability is dependent on what the animal does between the measurements. In particular if the animal is female and goes through a cycle of reproduction then the repeatability is considerably reduced. However, in female animals that do not reproduce it remains high even if the interval between measurements is >100 days (Duarte et al., 2010).

### Thermoregulation

**Figure 4:**
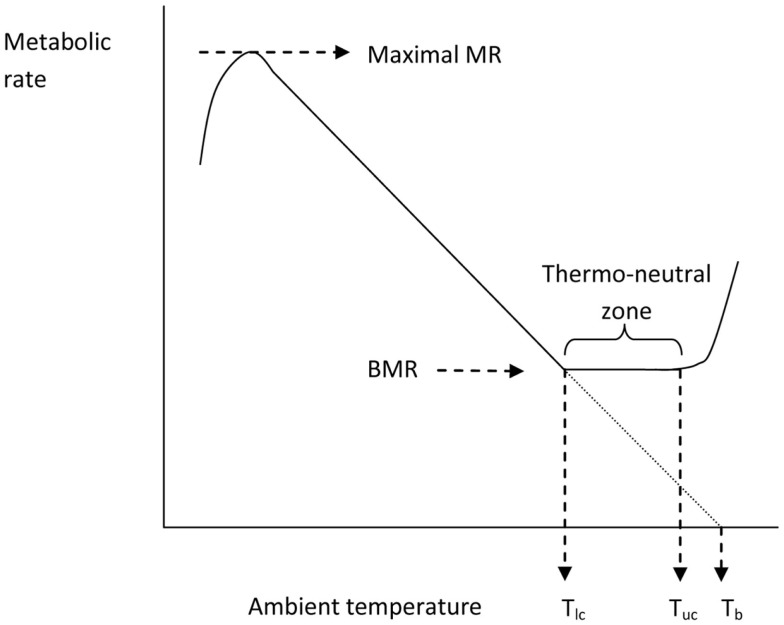
**The classical Newtonian cooling model describing the effect of ambient temperature on the metabolic rate of an endotherm like the mouse (after Scholander et al., 1950)**. *T*_lc_ = lower critical and *T*_uc_ = upper critical temperatures that bound the thermoneutral zone.

Issac Newton was among the first scientists to observed the temperatures of different sized bodies as they were cooled and warmed and kept in different ambient temperatures. The standard Newtonian model for the thermoregulatory response curve of an endotherm is shown in Figure 4. The expectation is that metabolism will increase at temperatures below *T*_lc_ in an almost linear fashion until the animal reaches a maximal metabolic rate. The gradient of this relationship between resting metabolism and ambient temperature is the whole body thermal conductance, and it extrapolates to the body temperature on the *x*-axis. Lower ambient temperatures than the temperature at which metabolism reaches a maximum lead to reduced metabolism because the maximum is unable to sustain body temperature and hence the reduced body temperature feeds back to reduce the metabolism until some dynamic equilibrium is reached. Above *T*_lc_ the BMR provides too much heat to balance thermoregulation requirements and hence evaporative water loss increases to dissipate this excess. At some point (the upper critical temperature) the animal must effect other mechanisms that paradoxically increase metabolism and lead to exponential increases in evaporative water losses and elevated body temperatures.

**Figure 5:**
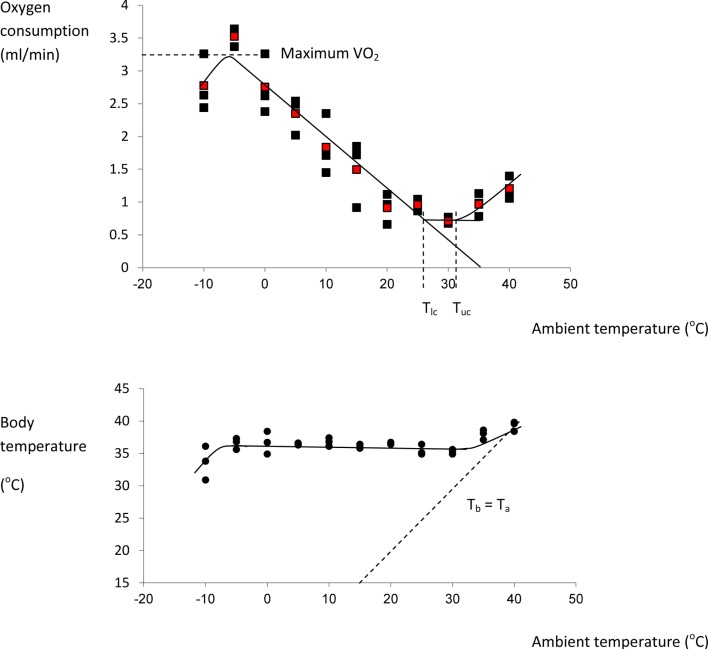
**Thermoregulation curve for the male MF1 mouse**. Individuals were measured for 3 h at 30 s intervals at each temperature in a single chamber-single analyzer system and the reported oxygen consumption was the lowest continuous 5 min period over the 3 h. Each point represents a different individual. Red points are means at each temperature. Body temperature (lower plot) was measured after the individuals exited the chamber. The characteristic temperatures bordering the thermoneutral zone (lower critical: *T*_lc_ and upper critical: *T*_uc_) are indicated on the upper plot, and the line of equivalence where body temperature (*T*_b_) equals ambient temperature (*T*_a_) is shown on the lower plot. (Data from Speakman, J. R., unpublished).

I could find no complete curves for mice in the literature although many incomplete curves have been published: Figure 5 shows the thermoregulation response curve for male MF1 mice, not acclimated to cold conditions. For MF1 mice this curve indicates a *T*_lc_ of about 26°C, and a maximal cold induced metabolic rate about 5.6× the basal metabolism attained at a temperature of −5°C. Based on this evidence mice seem to conform closely to the Newtonian cooling model. As noted above previous estimates of the lower critical temperature range between 26 and 30°C. These temperatures correspond closely to the temperatures that mice prefer (26–29°C) when given a choice (Gordon et al., 1998; Gaskill et al., 2009, 2012).

There are two basic mechanisms by which mice generate the heat to sustain their body temperatures below thermoneutrality. They use the heat generated by muscular contraction, i.e., they become physically active or they shiver, or they generate heat by non-shivering thermogenesis. Non-shivering thermogenesis is generally presumed to originate primarily in brown adipose tissue as a result of the action of uncoupling protein 1. The balance between different sources of heat is strongly affected by the animals previous history of cold exposure. In a naive animal exposed to the cold the response is almost completely from shivering. However, in animals that have been exposed previously to the cold for protracted periods the response is almost entirely due to non-shivering thermogenesis.

The curves in Figure 5 highlight that in normal laboratory conditions where mice are maintained at 19–21°C they are held under perpetual mild cold stress (4–6°C below thermoneutral). It has been suggested that keeping mice under these conditions may be a poor reflection of the situation in humans who live almost perpetually at thermoneutral temperatures (Swoap et al., 2008; Cannon and Nedergaard, 2009, 2011; Lodhi and Semenkovich, 2009; Overton, 2010; Karp, 2012). Housing temperature does seem to have an effect on some metabolic responses and genotype effects (see also Pincede et al., 2012 for the effects of ambient temperature on nociceptive tests in mice). For example in the UCP-1 KO mouse, studies at 21°C exposing them to a high fat diet did not reveal any phenotype relative to wildtype mice (Enerbäck et al., 1997) but these effects were potentially confounded by the background of the strains used. A later study revealed that when on a C57BL/6 background knocking out UCP-1 actually led to a paradoxical protection from diet induced obesity, which was absent at 26°C (Liu et al., 2003). Moreover, when these mice were maintained at 30°C they became obese relative to wildtypes, even when feeding on chow, an effect that was amplified when fed on a high fat diet (Feldmann et al., 2009). Although this demonstrates a strong effect of housing temperature, the reasons for the effect remain uncertain and opposite to that expected if UCP-1 is the main effector of non-shivering thermogenesis. Hence one might imagine its impact of its absence on weight gain would be greatest at the lower temperature since UCP-1 mediated metabolism contributes virtually nothing to BMR in mice at thermoneutrality (Golozoubova et al., 2001, 2006). Potentially other sources of non-shivering thermogenesis may be involved in these confusing responses of the UCP^−/−^ mice including for example from muscle mediated via sarcolipin (Bal et al., 2012). Given the responses to loss of UCP-1 span the whole range from protection from diet induced obesity at 21°C to susceptibility at 30°C, the question remains which of these responses most closely reflects the situation in humans. Speakman and Keijer (2013) compared the thermal response curves of mice and humans and concluded that for single housed mice the optimal temperature for comparison to humans would be around 23–25°C. At this temperature loss of UCP-1 seemed to have no impact on mouse susceptibility to a high fat diet induced obesity (Liu et al., 2003).

**Figure 6:**
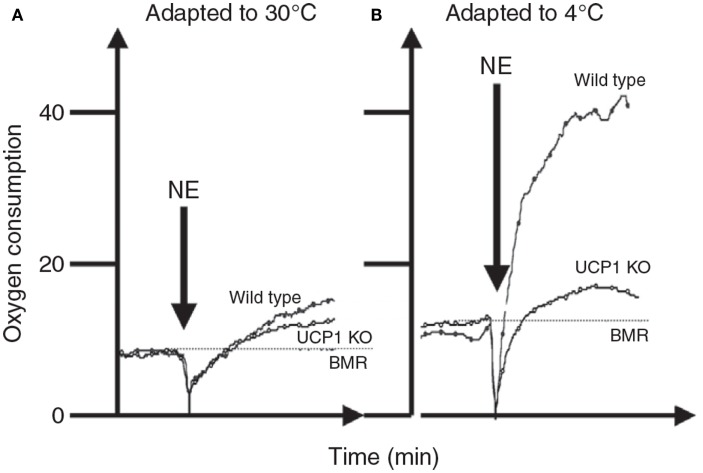
**The response of mice to norepinephrine injections**. In **(A)** the mice were maintained prior to the experiments at 30°C. There was only a very small response to the NE and it did not differ much between mice that have and do not have UCP-1. In **(B)** the mice were kept prior to the measurements at 4°C. Here the response to NE in the wild type mouse was much greater but that of the UCP-1 KO mouse similar to that in mice housed at 30°C. This suggests that all of the thermoregulatory conditioning to increase non-shivering thermogenesis by housing animals in cold conditions is mediated via UCP-1. Units for oxygen consumption were not stated and no time details were provided on the *x*-axis in the original source (data from Cannon and Nedergaard, 2011).

The whole body thermoregulation curve (Figure 5) cannot be used to generate an indication of brown adipose tissue or non-shivering thermogenesis as the heat to maintain body temperature is generated from multiple sources by multiple mechanisms. For the same reason it is also the case that acutely exposing an animal to the cold (e.g., 4°C) also cannot tell us anything much about its capacity for non-shivering thermogenesis (see also Cannon and Nedergaard, 2011). To measure non-shivering thermogenesis the procedure is generally to keep the mouse at a fixed ambient temperature (normally 30°C to prevent any shivering) and inject the mouse with noradrenaline to activate non-shivering thermogenesis via beta adrenergic receptors in the brown adipose tissue. Since beta adrenergic receptors are more widely distributed in the body and the dose required to stimulate the BAT is also sufficient to stimulate these other receptors there is some stimulation of non-shivering heat production in other tissues than BAT. Cannon and Nedergaard (2011) suggest this is purely a pharmacological effect that has no adaptive significance in the live animal. The extent of this non-BAT stimulation of metabolism by NA can be evaluated by comparing genetically manipulated mice with no UCP-1 to wild type mice with native UCP-1 (Figure 6). This suggests that for mice with no history of cold exposure almost all the heat produced following NA injection is from non-BAT sources, but for mice that had experience of cold previously, the contribution is much less at around 10–20%.

The procedure for the NA test of non-shivering thermogenesis is described in Cannon and Nedergaard (2011) and briefly summarized as follows. Animals can be measured awake (e.g., Jansky, 1973; Jackson et al., 2001a,b) but commonly they are first treated with a barbiturate based anesthetic. If a conscious animal is used the animal is placed into a respirometry chamber to obtain a baseline basal measurement (normally about 3 h: see above). Anesthetized animals show no physical activity and can be measured over a much shorter pre-injection period. The argument for anesthetizing the animals is that metabolic rate may be elevated as a stress response to injection in conscious animals. After the baseline measurement is complete the animals are then removed from the chamber and injected with NA by the dorsal subcutaneous route so that the injectate floods over the interscapular brown adipose tissue. IP injections generate a much poorer response. A dose response curve was produced by Heldmaier (1971) which suggested doses over 1.0 mg/kg elicit a maximal response. Doses higher than 1.5 mg/kg can be fatal (pers. obs.). The animal is then immediately returned to the respirometry chamber. Normally, awake animals remain completely quiescent after the injection. This is probably because any physical activity would exacerbate the induced heat production and make them at risk of fatal hyperthermia. Since stressed animals would normally manifest their stress by elevated physical activity the presumed impact of stress in the measurement of NA-induced metabolism in conscious animals has probably been overemphasized.

**Figure 7:**
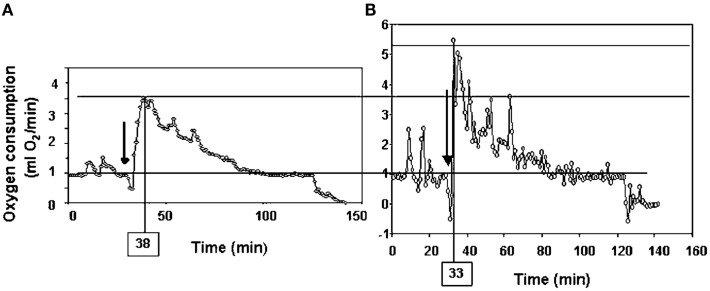
**Time course of the response of a small rodent (the short-tailed field vole: *Microtus agrestis*) to injection of Norepinephrine**. The plot in **(A)** is the raw data from the respirometry chamber. In **(B)** the data have been mathematically manipulated to reconstruct the instantaneous changes in metabolism (figure from Arch et al., 2006 and original data from study by Jackson et al., 2001a).

**Figure 8:**
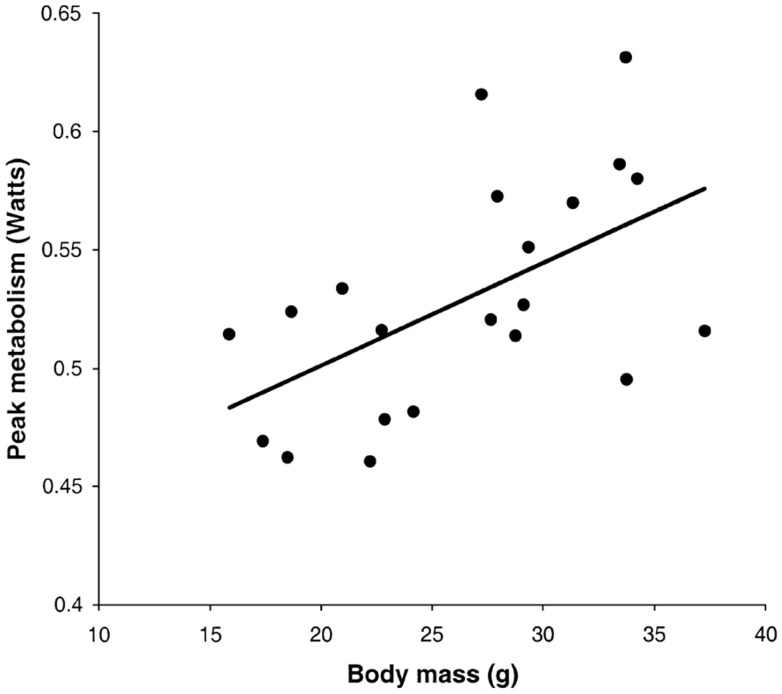
**Instantaneous peak metabolic rate following norepinephrine injection in relation to body mass**. Data are for a small rodent about the same size as a mouse (the short-tailed field vole: *Microtus agrestis*; Jackson et al., 2001b).

Following return to the chamber there is a large increase in metabolic rate which reaches a peak and then subsides – a typical example is shown in Figure 7A (Jackson et al., 2001a; Arch et al., 2006). The shape of this curve depends completely on the chamber characteristics in which the measurement is made. In a fast washout system the peak reached will be much higher than in a slow washout system. Comparing peak responses across studies is therefore complicated by lab specific details of the respirometry systems utilized. There are methods to get over this problem discussed in more detail below in the context of measuring physical activity costs. The results of applying such a conversion to the data in Figure 7A is shown in Figure 7B. These data show that even when a fast washout small volume chamber is used the “instantaneous” estimates of metabolism can still be substantially higher than the actual measurements if the metabolism is changing rapidly. In this case the difference was 40%. The area under the curve is a chamber independent measure of the response that does provide a possibility for comparisons but is generally never reported as measurements are frequently discontinued before the metabolism returns to baseline. The instantaneous peak response to NA injection is strongly dependent on body mass (Figure 8; Jackson et al., 2001b) which means the body mass effect must be taken into account when comparisons are made between different genotypes (see below under analytical considerations). Attempts to quantify the NST activity in response to NA using infrared thermography to quantify the surface temperature rise above the iBAT have been attempted (Jackson et al., 2001b) with limited success.

### Physical activity

Mice have a range of physical activities but their primary mode of locomotion is running. The cost of running in mice can be measured using a tread-wheel apparatus within the respirometry chamber. This allows the speed of running to be manipulated by the experimenter and the consequent costs of locomotion at each speed derived. The main requirement in such procedures is that the animal reaches a steady state performance of the behavior for a long period relative to the washout characteristics of the chamber. That is the animal needs to run continuously for several minutes so that a stable running metabolic rate can be measured. Mice in captivity (and probably also in the wild) seldom run for such protracted periods so their behavior may not always be adequate, and some training in the apparatus is normally necessary before the animals will perform the required behavior. We have found that varying the speed during training seems to improve the behavior, perhaps because mice normally oscillate the speed at which they move.

**Figure 9:**
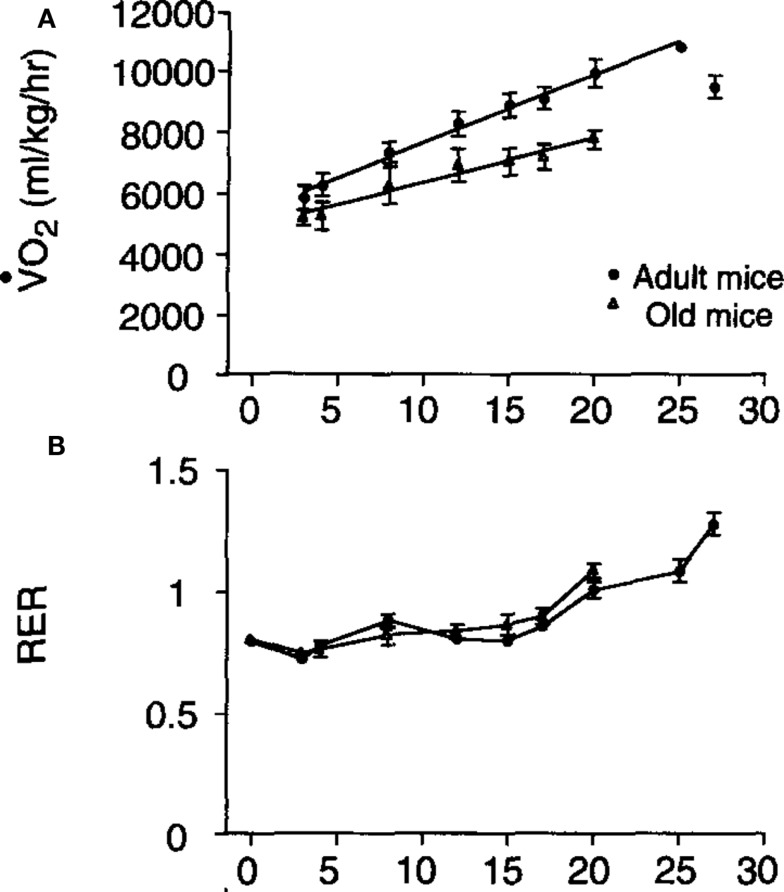
**(A)** Mass specific oxygen consumption in relation to running speed for adult and aged mice and **(B)** the patterns of respiratory exchange ratio (RER) in the same animals (from Schefer and Talan, 1996).

The relationship between running speed and metabolic rate in mice is linear (e.g., Figure 9: Schefer and Talan, 1996). Extrapolating the relationship back to the *y*-axis at a running speed of zero generally yields a value that exceeds the measured rate of basal metabolism. In the example shown in Figure 9 the extrapolated *y*-axis intercept was between 5,000 and 6,000 ml/kg/h but the actual measured resting metabolic rate was between 2,700 and 3,200 ml/kg/h. This difference has been often interpreted as a “postural” cost of locomotion. The data in Figure 9 also illustrate that the cost of locomotion depends on subject age and that the RER is also dependent on running speed with higher speeds being associated with elevated RER values. In this case the division of the values by body mass could mean the age effect was an artifact of a body mass difference (see below under analytical considerations), but in fact the aged mice were lighter than the adult mice so this age effect was not an artifact.

More often, however, rather than the costs of locomotion, researchers are interested in how much of the daily energy budget of a mouse can be ascribed to “general” physical activities. This might include “locomoting” but would also include many other behaviors such as grooming, climbing on the cage bars, and eating, etc. Ascribing a cost to this sporadic data from chamber studies is difficult for two reasons. First, the behaviors are highly variable and most likely have different costs. Second, if a mouse performs a behavior within a metabolic chamber the record of its metabolic rate by the analyzer is not an instantaneous reflection of the actual metabolic rate. There is the mixing in the chamber to be considered plus the delay between the excurrent gas exiting the chamber and arriving at the analyzer. Thus the peak metabolic rate measured following a behavioral event is a poor reflection of the actual costs of the activity. Probably the first study to consider these issues was that by Bartholomew et al. (1981) who studied the warm-up metabolism in moths. They realized that the observed metabolic measurements could be used to reconstruct the actual time course of metabolism if information on the washout characteristics of the chamber being used were known, and the change in metabolism between measurements was used in addition to the actual measurements. This would enable reconstruction of the “instantaneous” estimates of metabolism. The procedure is known as deconvolution, and full details of it can be found in Arch et al. (2006) and Lighton (2008). The effects of applying this approach on the metabolism curve following injection by NE in Figure 7A are shown in Figure 7B. These data show that the “actual” peak metabolic rate was 40% higher than the highest measurement in the chamber and occurred much earlier.

An example of using this methodology was the study by Speakman et al. (1989) to measure the energy costs of echolocating behavior in small bats. By converting the actual measurements to the equivalent “instantaneous” estimates of metabolism it was possible to regress the echolocation behavior of the bats (pulses/minute) on the metabolic rate to work out the cost of echolocating. This still did not take into account of the lag between the excurrent flow leaving the chamber and being measured at the analyzer, so to account for this the regressions were performed stepping the metabolic rate measurements relative to the behavior measurements. This showed the maximal *r*^2^ for the regressions corresponded to a lag of about 2 min, approximately corresponding to the expected lag based on the flow rate and system configuration.

In this latter application the behavior was very simple to correlate against the instantaneously corrected metabolism because the behavior could be easily characterized in numbers (echolocation pulses). For mouse behavior this is more problematical but fortunately a solution to the problem of characterizing mouse behavior as activity has been produced and this involves monitoring the movements of the animals and then converting these movements into “counts.” There are different proprietary solutions to this problem based on different technologies for monitoring the movements and the data they generate is not equivalent. However, if the behavior of an animal is monitored while it is in a respirometry chamber and it is converted into “counts,” then it would be a relatively straightforward matter to regress these counts onto the derived estimate of “instantaneous” metabolism in the same way as performed previously to estimate the cost of echolocation. In fact this has not yet been done, but instead some studies have regressed the counts of activity onto the “simultaneous” uncorrected metabolic rate estimates (e.g., Bjursell et al., 2008). The reason for this is because the systems used to perform this work have been switching systems where each chamber is monitored relatively infrequently, the chambers are large and the washout is relatively slow. Hence the refinement of making “instantaneous” estimates of metabolism cannot be performed, and the lag of the system is small (seconds) relative to the time between measurements of each chamber (minutes). The resultant regression is used to estimate the costs of activity (gradient of the regression) and the RMR (intercept; e.g., Nonogaki et al., 2003) from data spanning 24 h or longer periods. One potential issue with this approach is that it assumes the baseline RMR is constant, yet we know that RMR will vary depending on the time of day (active and quiescent phases) and also on the thermic effect of food.

To overcome this issue a much earlier study of rats by Even et al. (1991) used an approach called Kalman filtering to reconstruct the varying baseline RMR in a situation where the rats were moving around freely in the respirometry chamber. This method has subsequently also been applied to mice (Deveaux et al., 2009). Full details of the approach are in Even et al. (1991). A potential issue, however, is that Kalman filtering requires a more frequent sampling of the metabolism than is generally available from the use of multiple chambers linked up to switching devices. van Klinken et al. (2012) devised a penalized spline regression method to attempt to reconstruct the time varying RMR and showed that with a sampling frequency of 10 min this provided an estimated time dependent RMR that was 1.7× more accurate than using the Kalman filtering approach, and 2.7× better than linear regression. However, a 10 min sampling interval would be a fast turnaround time in a multi-chamber switching device, and the estimated RMR became systematically less accurate as sampling time increased above 10 min. The relative standard deviation in the estimated activity costs was similarly very sensitive to the sample time. At present reconstructing activity costs from these chambers results in estimates that have poor accuracy (Even and Nadkarni, 2012) – although the situation is constantly evolving.

### Bicarbonate method

An alternative approach to measuring the costs of physical activity in mice is to use an isotope based technique called the labeled bicarbonate method (Hambly and Voigt, 2011). In this method the rate of CO_2_ production is measured by injecting animals with a bolus dose of ^13^C labeled sodium bicarbonate. This comes to rapid equilibration (<5 min) with the body bicarbonate pool and is then eliminated from the body exponentially in relation to the rate of CO_2_ production – hence providing an indirect measure of metabolism (Hambly and Voigt, 2011). Because the label appears in expired CO_2_ it can be easily and non-invasively applied by measuring breath samples. This technique has been primarily used to measure the energy demands of unencumbered flight in both birds (Hambly et al., 2002, 2004a,b), and bats (Voigt and Holderied, 2012; Voigt and Lewanzik, 2012). However, an earlier study was performed in mice to validate the method against indirect calorimetry (Speakman and Thomson, 1997) and it seems to give a reasonable estimate of metabolic rate over periods of 30–90 min. This could in theory be used to measure the energy demands of activity by monitoring what animals do over the measurement period and then assessing costs across several individuals using multiple regression techniques.

### Daily energy expenditure

Although multi-chamber switching devices are relatively poor for the determination of the components of metabolism, especially BMR (see above), these machines really come into their own when faced with the issue of measuring long term energy demands like the daily energy expenditure. This is because in this application the fine time resolution needed for an accurate estimate of BMR is unnecessary, and a chamber is required that mimics as closely as possible the home cage environment. This is impossible to achieve using the sorts of Spartan low volume chambers that are necessary to accurately determine BMR where there is often no capacity to also provide the animals with food and water. Multichannel systems using large chambers have become increasingly sophisticated with the measurement chamber also being instrumented with sensors to monitor ambient temperature, physical activity levels, food, and water intake and body mass of the subject. With automatic baseline measurements it is feasible to leave animals in these chambers for periods of several days to obtain repeated measures of the total daily energy expenditure. In this circumstance having a measurement every 20 min or so is adequate to evaluate the total daily energy demands, and the slow washout characteristics that are consequent of having a large chamber relative to the flow rate, and a complex chamber design that further reduces the washout time is actually an advantage because this makes the sampled time point more likely to reflect an average over the more protracted period of metabolism. Several excellent machines in this respect are available the main ones being the CLAMS system produced by Columbus instruments, the Phenomaster system produced by TSE systems, Ltd., and the Promethion system by Sable systems, Inc. These will all provide an accurate estimate of DEE. If you require to decompose the metabolic rate into resting and active components algorithms are currently in development by the manufacturers to achieve this (see van Klinken et al., 2012) but they are currently insufficient to achieve the sorts of accuracy that is possible using a single analyzer-single chamber system and a small volume chamber (Even and Nadkarni, 2012). The exception to this may be the Promethion device which also uses single chamber-single analyzer approach that can then be analyzed using the Kalman filtering method advocated by Even et al. (1991) or the penalized spline method by van Klinken et al. (2012). However things are currently moving rapidly in this field and in future accurate decomposition of the total daily energy demands into the main two components (rest and activity) may be feasible. At present, however, the best advice would be to use these devices to get good estimates of DEE, but use single chamber-single analyzer systems to obtain specific components such as BMR and RMR.

**Figure 10:**
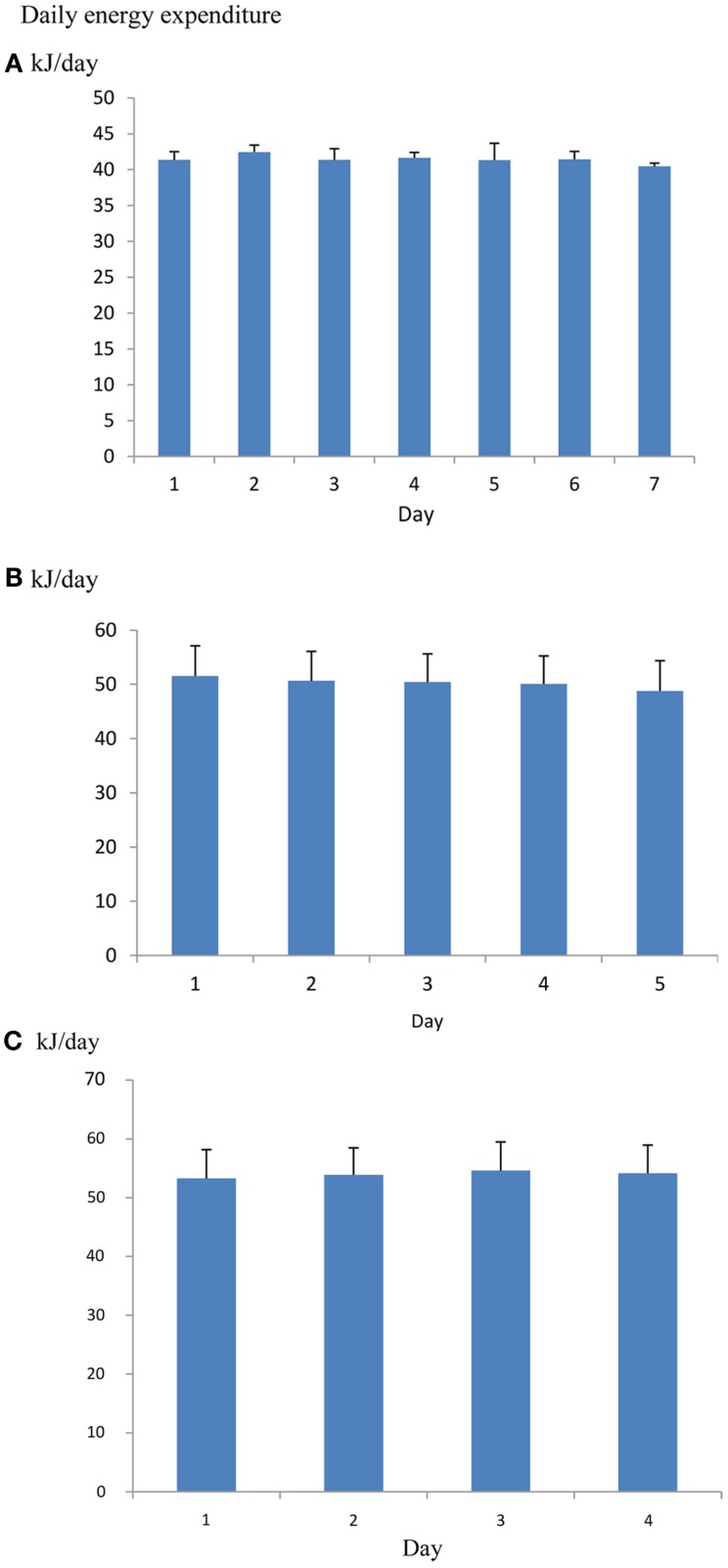
**(A)** Mean daily metabolic rates (kJ/day) of four mice living in an Oxymax (CLAMS) open flow respirometry system continuously for 7 days. Error bars are standard deviations. Mice were placed into the system 2.5 h before recording started. There was no significant change in metabolic rate over the 7 days (Data from R. Sinclair and J. R. Speakman, unpublished). **(B)** the same type of data for 16 mice living in a TSE phenomaster system for 5 days (data from Timo Muller, pers. comm.) and **(C)** 15 mice living in a Promethion system for 4 days (data from Brent Wisse via John Lighton, pers. comm.). There was also no significant change over the 5 and 4 days respectively.

One issue when using such systems is how many days the animal should be left in the chamber to provide a useful measurement. It is common practice to discard the first day since this may be contaminated by exploratory behavior in the novel environment and then leave the animals in the system for 5–8 further days of measurement. Some preconditioning to the system may also minimize the novelty effect (Tschoep et al., 2012). However, if mice are placed into the chamber a couple of hours before recording begins there is no significant effect of day over 7 days of measurement (Figure 10), suggesting that rejecting the whole first day of measurements may be overly cautious. The overall coefficient of variation (overall SD/overall mean) across repeated measurement days is about 3% (calculated from data in Muller et al., 2013). Hence averaging the metabolic rate across five consecutive days would yield an average estimate of DEE with a 95% confidence interval of also ±3% around the mean.

### Measuring DEE for animals in social situations

In some situations measuring the daily energy expenditure of a mouse is impossible by the standard methods of indirect or direct calorimetry. These include for example the measurement of a female mouse when she is lactating. Measuring BMR of such a mouse can be performed (e.g., Johnson et al., 2001; Krol et al., 2003; Krol and Speakman, 2003a; Zhao et al., in review) by separating the mother from her pups and putting her into the chamber alone. This works for a BMR or RMR measurement, although there are some special considerations to made. Mice separated from their pups tend to be more active and take longer to settle down. In these circumstances a four rather than a 3 h standard measurement may be necessary. In addition lactating mice are often active and feed during the day. If they are food deprived for 4 h prior to the measurement followed by a 4 h measurement without food (e.g., Zhao et al., in review) this may potentially have an adverse impact on their lactation performance.

However, if a DEE measurement over 24 h is required then clearly separating the mother from her pups for this length of time would be impossible, yet the mother cannot be placed into the chamber with her pups because the resultant estimate is the summed energy expenditure of the combined mother and pups, not the mother alone. Other situations involve similar issues – for example measuring the energy demands of a single mouse when it is embedded in a social situation. For example, studies have been made of the consequences of social defeat on energy balance in mice (Bartolomucci et al., 2009). When a dominant and a subordinate mouse are housed together the dominant mouse appears resistant to weight gain but the subordinate mouse is not. These differences may be rooted in differences in their daily energy expenditure, but clearly separating the mice to measure them removes them from the paradigm that generates the difference we are trying to measure.

In these situations an alternative approach is needed. Two such approaches are the doubly labeled water technique and the heart rate technique (Butler et al., 2004). The heart rate method relies on the fact the fluctuations in energy demand are generally met by variations in heart rate. Hence it is possible to construct an individual calibration between energy metabolism and heart rate using standard indirect calorimetry with the animal in the chamber alone and then reconstruct the time course of energy demands over 24 h by logging the heart rate of the animal later when it is engaged in its social activities. This method has been used widely to measure the energy demands of free-living animals, but I am not aware of its application to date in the mouse. Technologically it is feasible because heart rate loggers capable of being implanted into mice are currently available (e.g., from DSL, Ltd., and from Minimitter).

The other technique, the doubly labeled water technique, is an isotope based method that relies on the differential elimination of isotopes of hydrogen and oxygen from the body (Speakman, 1997). Oxygen isotopes in the body water are eliminated by the dual flux of water and CO_2_ through the body, while hydrogen isotopes are eliminated only by water. Hence the magnitude of the difference in the elimination of the isotopes is directly related to the CO_2_ production, and particularly if RQ is known, the energy metabolism. This method was actually developed in the 1950s in mice (Lifson et al., 1955). It has been subsequently refined, and the refinements validated in comparison to indirect calorimetry using voles (Speakman and Krol, 2005). This refined method has been applied in multiple studies in particular to measure the energy demands of lactating female mice (Johnson and Speakman, 2001; Johnson et al., 2001; Krol and Speakman, 2003b; Krol et al., 2007; Zhao et al., in review) and other small rodents (Wu et al., 2009; Simons et al., 2011).

Although these two methods come into their own when mice are in social systems and cannot be measured by indirect calorimetry, there is no reason why such methods could not be used in mice more generally to measure their energy demands over 24–72 h using DLW or much more protracted periods of days and weeks using the heart rate approach. Their complexity, for example requiring mice to undergo surgical procedures for the heart rate method, and the requirement for expensive mass spectrometry equipment for the DLW method, has probably inhibited their use to date.

## Analysis and Presentation Issues

### Detecting effects of genotype

One of the commonest analytical situations in the study of mice is when one wishes to detect the impact of a genetical manipulation on the rate of energy expenditure. On the face of it this is a simple issue. One would measure a sample of mice representing each genotype and then compare their rates of energy utilization (Watts) using standard statistics such as the *t*-test or Analysis of variance (ANOVA). The problem is that generally when there has been an impact on the energy demands this is translated into a difference in body weight. Body weight is one of the key factors driving the rate of energy expenditure. Bigger animals have more metabolizing tissue and expend more energy. Hence if a difference is detected in the rate of energy expenditure this may be a secondary effect of the altered body weight, rather than a primary effect of the genotype alone.

**Figure 11:**
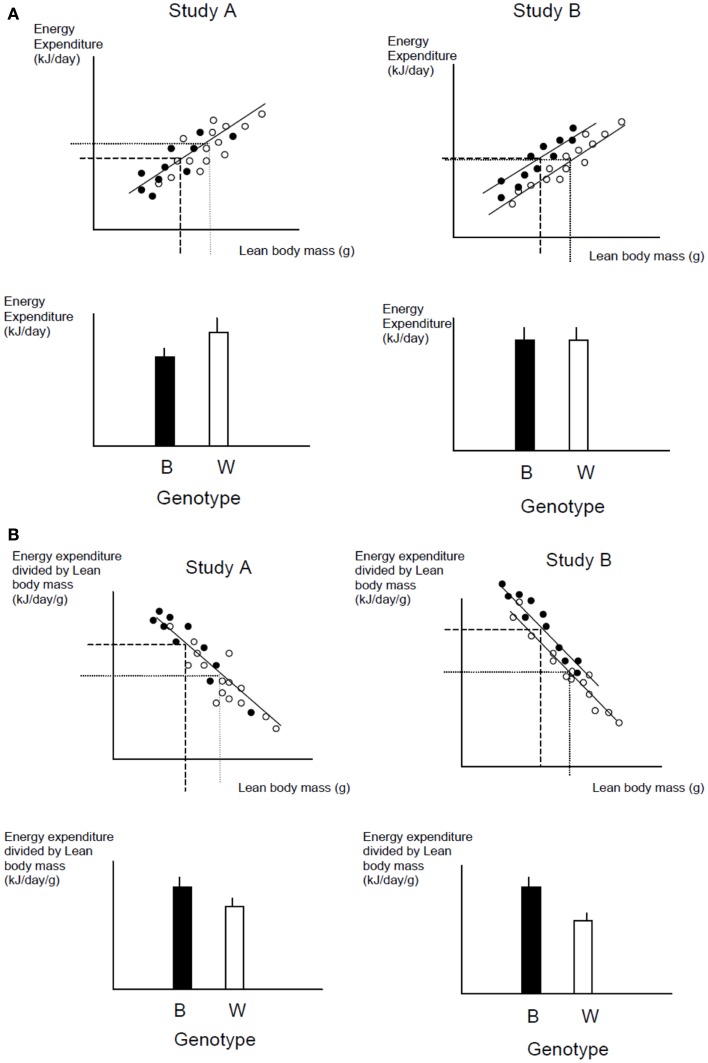
**(A)** Hypothetical data from two studies of two different genotypes (black and white). In both studies there is a lean body mass difference between the two genotypes. In study **(A)**, however, the data for energy expenditure lie on a common line in relation to lean body mass. There is no difference in their energy expenditure apart from an effect due to lean body mass. In study **(B)**, the data for expenditure lie on two separate lines relative to lean body mass. In this situation, there is an effect of the genotype on expenditure independent of any mass effect. The challenge is to find an analysis that separates these two situations. If we use the raw data and average across the individuals for each genotype, the results shown below the plots as histograms reveals that there is a significant difference in study **(A)**, with energy expenditure of the black genotype being lower than that of the white one, whereas in study **(B)**, there is no significant difference in energy expenditure between the genotypes. To see if there is a genotype effect on expenditure independent of any effect of lean body mass we may divide the energy expenditure by BW **(B)**. The result in study **(B)** now reveals that expenditure in the black genotype is higher than that in the white genotype. However, this division also reveals a significant effect in study **(A)**, where none actually exists. The problem is that the division by lean mass overcompensates for the mass effect.

A frequently used approach to try and rectify this effect is to simply divide the energy metabolism by the body weight to generate values of energy expenditure/gram (Watts/g). Butler and Kozak (2010) highlighted 10 very high profile papers in the top scientific journals where this method had been used, and Tschoep et al. (2012) reviewed over 50 articles on energy metabolism in mice and found that this approach had been used in almost 70% of them. This approach, however, only normalizes for the effect of body mass when the intercept of the relation between metabolic rate and body mass is at the origin. In the case of measurements of energy metabolism this is seldom the case. The reason why such relationships do not normally pass through the origin is because mice are made up of different tissues that metabolize energy at very different rates. In particular *in vitro* estimates of the energy metabolism of fat and skeletal muscle are substantially lower than for tissues like the liver, kidneys, heart, and brain (Krebs, 1950; Elia, 1992). When an animal grows larger it generally does not grow each of its tissues in direct proportion to each other (isometrically), when it loses weight it will generally draw more on adipose tissue than lean tissue, and differences between strains or genotypes also include changes in the ratio of fat to lean mass in addition to total body weight. Hence in most circumstances that researchers are interested in differences in weight are paralleled by differences in composition. Consider therefore the following simple example (after Speakman et al., 2002). If a 40 g mouse of strain A consisted of 30 g of lean tissue and 10 g of fat, and the lean tissue expended energy at 30 mW/g and the fat tissue expended energy at 10 mW/g, the total metabolic rate would be 1 W (30 × 30 + 10 × 10). The energy expenditure/gram of body weight would be 25 mW/g. If there was a mouse from a second strain B that had the exact same tissue metabolic rates (30 mW/g for the lean tissue and 10 mW/g for the fat tissue) but in this case the mouse weighs 50 g, comprising 30 g lean tissue and 20 g fat tissue, its total metabolism would be 1.1 W (30 × 30 + 20 × 10; 10% higher). The whole animal metabolic rate/gram of body mass would fall to 22 mW/g (12% reduced compared to strain A). Dividing by body weight in this situation therefore creates the spurious result that the metabolic rate of the heavier and fatter strain B mouse is lower, when in fact the metabolism of each of its tissues is identical to the strain A mouse. One may equally imagine a situation where the energy metabolism of the lean tissue in the lighter mouse (strain A) was 33.3 mW/g and that in the larger mouse (strain B) was 30 mW/g (an 11% lower metabolic rate), but in this situation dividing by weight would result in no difference between the two mice. Dividing by weight may therefore create spurious effects or alternatively mask real effects, but will almost never give the correct answer (Packard and Boardman, 1987; Allison et al., 1995; Poehlman and Toth, 1995; Himms Hagen, 1997; Arch et al., 2006; Butler and Kozak, 2010; Kaiyala and Schwartz, 2011). A graphical illustration of the problem is shown in Figure 11.

Recognizing that the intercept of the relation between energy expenditure and mass is seldom zero a different approach has been to divide by mass raised to some power <1.0 and >0.0. The value of choice differs between studies (commonly used powers are 0.75 and 0.66 (10 and 5% of studies reviewed by Tschoep et al., 2012, although occasionally other values are used – e.g., 0.83 Austad and Kristan, 2003). The source of these values are the fitted scaling exponents for the relation between mass and energy expenditure across species (Kleiber, 1961; White and Seymour, 2004, 2005). The assumption here is that changes in body composition across species as one moves from mice to elephants are similar to those as one moves from a small mouse to a larger one. This is unlikely to be the case. Nevertheless such an approach may occasionally by chance hit on the correct answer, if the inter-specific and intra-specific gradients coincide. It is, however, largely a chance effect. Sometimes it will be correct and other times not, and in yet other cases it will generate a result when none exists, in the same way dividing by mass alone can do as illustrated above. The problem is we never know which case we are dealing with. In the example above if we employ the commonly used inter-specific scaling exponents, then metabolism divided by mass raised to the 0.75 power results in a metabolic rate of 62.9 kJ/g^0.75^ for strain A and 58.5 kJ/g^0.75^ for strain B (a decrease of 7%). Using the other commonly used scaling exponent of 0.66 gives values of 87.6 kJ/g^0.66^ for strain A and 83.2 kJ/g^0.66^ for strain B (a decrease of 5%).

**Figure 12:**
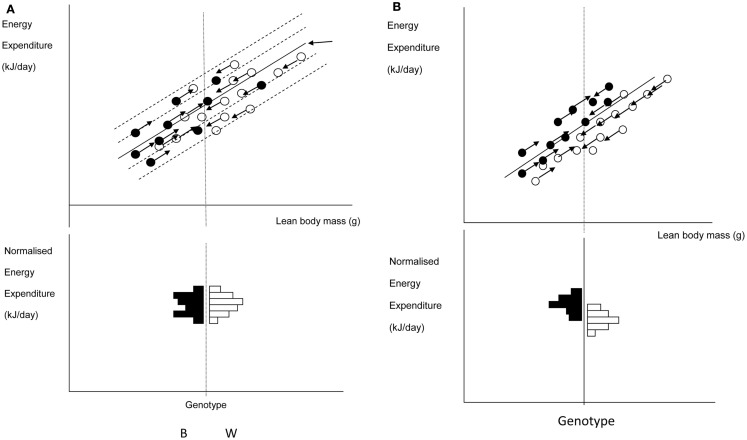
**This figure illustrates the data from the two hypothetical studies (A,B) shown in Figure 11 to illustrate the mechanism by which ANCOVA normalizes data**. ANCOVA works by effectively fitting a gradient to the data and then sliding all the individual data points along imaginary parallel lines until they all group together at the average lean BW. This creates two distributions that can then be tested to see if they differ from each other. This approach is illustrated below the first panel, with the black genotype on the left of the overall mean and the white genotype on the right. As can be seen there is no significant difference. If we repeat this process for study **(B)** sliding the data down the imaginary gradients yields a different result in that the two distributions are now separated.

There is however an accepted statistical solution to this issue called analysis of covariance (ANCOVA). In effect what ANCOVA does is rather than assume a gradient for the relationship between mass and energy demand, it fits a gradient to all the actual data. It then uses this individually tailored gradient to remove the effects of mass, and asks if there is any remaining effect of the genotype. How it does this is really simple. First a gradient is fitted to the data using regression techniques. A vertical distance is then calculated from each data point to the fitted line. These values are called the residuals to the fitted regression. The residuals are then compared between the two genotypes, taking into account the degree of freedom that is used for fitting the regression gradient to the data. An example of this process using the hypothetical data set in Figure 11 is shown in Figure 12 (from Tschoep et al., 2012). This is an extremely powerful solution because it makes no assumptions about the gradient but rather fits an empirical gradient to each data set. Unfortunately this approach has been used in <2% of the studies where an effect of genotype on metabolism has been examined (Tschoep et al., 2012). Examples of the use of this method are Speakman and Racey (1991) to compare the metabolic rates of echolocating and non-echolocating bats, and in mice the studies of Meyer et al. (2004) and Claret et al. (2007).

**Figure 13:**
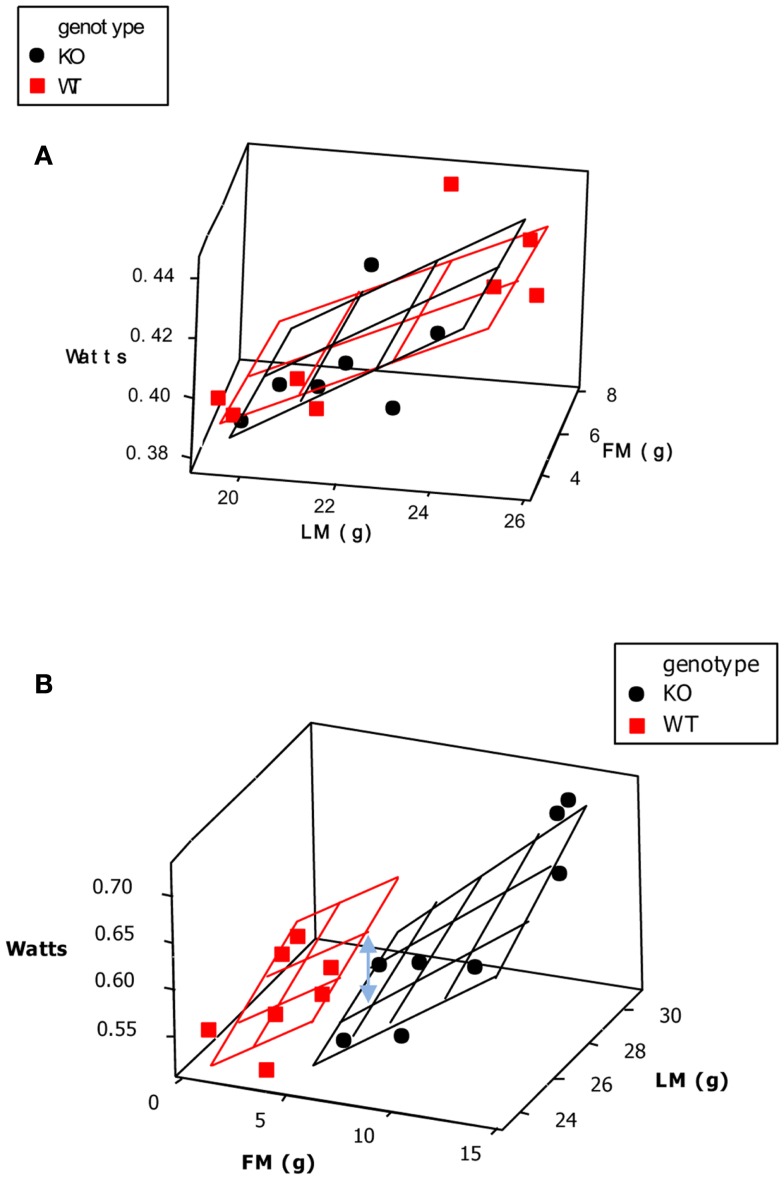
**3D plots showing the effects of tissue specific knockouts of the p62 gene on energy expenditure taking into account the effects of lean (LM) and fat mass (FM)**. Wildtype mice are shown as black dots while the KO mice are shown as red squares. The black and red planes respectively represent the best fit regression models that fit the data (based on general linear modeling). In both plots the *y* variable is energy expenditure (Watts) and the *x* and *z* variables are the body component weights in grams. In **(A)** (p62 muscle specific KO) there is no difference between the planes that best describe the data using general linear modeling but in **(B)** (p62 BAT specific KO) there is a systematic reduction in the metabolism of the KO animals, illustrated as the difference between the planes (blue double headed arrow). In both cases the initial 3D plot was generated using the statistics program MINITAB and the planes were then manually added using values from the regression equations to fix their locations (from Muller et al., 2013).

The example in Figure 12 also makes a different, but equally important point, and that is the problem of presenting energy expenditure data as histograms. Histograms which show raw metabolic rates (Watts), or metabolic rates divided by body mass (or by fat-free mass: see below) do not provide any information about what is happening in the relationship between metabolism and body mass (Tschoep et al., 2012). Hence they do not provide the necessary information to evaluate what is going on. A much better approach in the presentation of these effects is to show the plot of the relationship between mass and metabolism, and if necessary add a histogram of residuals to this to emphasize the significance of the genotype effect (Figure 13). Tschoep et al. (2012) recommended the use of raw histograms to present these types of data should be phased out.

Since the issue with differences in body mass comes about because of the change in body composition then it has been argued that perhaps a better solution is not to use ANCOVA but to simply divide the metabolic rate by the mass of the metabolizing tissue (Butler and Kozak, 2010). Several devices are available which allow the *in vivo* measurement of fat mass and fat-free mass in mice (such as DXA, MRI, and CT scanners). Since the *in vitro* metabolic studies point to a major difference in the metabolic rates of lean and fat tissue then it has been argued that dividing by the fat-free mass is a preferable alternative to ANCOVA (Butler and Kozak, 2010). It is the second most popular method for “normalizing” energy expenditure data currently in use in the literature. If it was correct that all of the metabolism was attributable to the lean tissue mass then this would indeed be a potentially valid approach. However two things undermine this claim. The first is that lean tissue is itself not homogenous, and the different components of lean tissue do not scale isometrically with total body mass, hence the relation between lean tissue mass and metabolism is also unlikely to pass through the origin – as required for a simple ratio to be used. The second problem is that while fat tissue appears to have a very low metabolism *in vitro* this does not correspond to its apparent metabolism *in vivo*. When multiple regression data are fitted to metabolic rates with lean tissue and fat tissue mass as predictors then the effect of fat tissue is often about 1/3 that of the lean tissue (Johnstone et al., 2005; Kaiyala et al., 2010). This is about 10× higher than the expectation based on *in vitro* estimates (Krebs, 1950; Elia, 1992). This is probably not because fat tissue becomes suddenly metabolically active when it is in a living body, but probably because it secretes adipokines that stimulate lean tissue metabolism. Leptin is strongly implicated in this effect (Kaiyala et al., 2010).

In the same way as dividing by mass to the power 0.66, or to the power 0.75, dividing metabolism by fat-free mass (or lean body mass: LBM) may by chance generate the correct answer. It is more likely to be the correct answer than dividing by total body mass. Nevertheless, it is also potentially the wrong answer. In the example we detailed above dividing the metabolism by lean tissue mass gives estimated metabolic rates of 33.3 mW/g LBM for strain A and 36.7 mW/g LBM for strain B – in a situation where tissue metabolic rates were actually identical. The important point is that there is no actual need to take this risk. If information is available on fat and fat-free masses of the individuals involved in the measurements then it is possible to include these two continuous variables in the ANCOVA as independent predictors of metabolism. In effect instead of fitting a simple regression model (to body weight) and calculating the residuals to this relationship, one is fitting a multiple regression model with fat and fat-free mass as predictors and then calculating the residuals to this multiple regression.

Although ANCOVA is a powerful method for analyzing these types of data it is important to recognize that it comes with its own set of assumptions and in certain circumstances will not work effectively. The first such situation worth considering is where the relationships between metabolic rate and mass (total, fat, or fat-free) are non-linear. This can normally be spotted when plotting the data as a bivariate plot and the problem can be overcome by transforming one or other of the variables. The second problem is also easily recognized from the mass-metabolism plot but is less easily overcome. This is the situation where there is absolutely no overlap in the data from the two genotypes on the mass axis. In this circumstance the variance explained by fitting two lines through the data, as is performed by ANCOVA, does not generate a significantly lower residual variance that fitting a single gradient through both data sets, leading to a non-significant genotype effect – but this may be an artifact of the data not overlapping. A solution is to calculate the regression parameters within each data set independently and then manually adjust the data using these relationships to a body mass mid way between the data sets and make the comparison at the position using a *t*-test. However, this requires extrapolation of the data beyond the limits of each data set and the resultant adjusted estimates may have too large confidence intervals to be useful. Even and Nadkarni (2012) suggested a solution to this problem when the assumptions of ANCOVA are violated would be to calculate a “metabolic equivalent weight” as the lean body mass +0.2× the fat mass, based on the fact lean body mass has approximately 20% the metabolism of lean body mass *in vivo* (after Arch et al., 2006). This remains an interesting but untested suggestion. A final issue with using fat-free and fat mass as predictor variables is how to present the data. Ideally this is done as a 3D plot with metabolism as the *y*-axis and *x* and *z* axes being fat and fat-free mass respectively. Examples of such plots for a significant and a non-significant effect of a genotype are shown in Figure 13 (from Muller et al., 2013).

### Required sample size and power analysis

Another argument made by Butler and Kozak (2010) regarding the superiority of dividing by Fat-free mass as opposed to using ANCOVA is that the sample size required for ANCOVA is much larger. This is a spurious argument because simply dividing by mass is easier to calculate but does not generate any greater ability to separate two sets of data. This does however raise the interesting issue of required sample sizes for such studies. Conventional inferential statistics based on probability testing are designed to minimize the risk of a type 1 error. That is wrongly inferring something is happening when it is not. This is because studies have historically been less concerned about making type 2 errors. That is failing to spot an effect when one actually exists. However, in terms of diagnosing the effect of different genes, a type 2 error is as serious as a type 1 error. Conventional probability testing only tells us half the story (the risk of a type 1 error). What we actually also need to know is how confident we can be when we say there is no effect that we are not making a type 2 error. This is done by power analysis.

When power analysis is done in advance of an experiment being performed it can be used to establish the sample size of individual mice required in each of the genotype groups to be 95% confident we are not making a type 2 error if we find no significant effect. To do this we have to decide how big an effect would be important from a biological standpoint. For studies of energy metabolism this could be quite small (i.e., about 3–5%) as we often infer small effects on energy intake or expenditure accumulated over time may ultimately cause large effects on body composition. This is the ideal way to use power analysis. However it can also be used another way and that is to do a *post hoc* power analysis. This tells us the power we have to say there really is no significant effect of the magnitude we have detected, given the variances and sample sizes of the data sets for the two genotypes.

Using power analysis can be a very sobering exercise because it reveals that virtually every study performed to date to diagnose the effect of a given gene on energy intake has been insufficiently powered to detect the small effects in energy balance that might be biologically important. Tschoep et al. (2012) calculated that for a typical study of energy intake one might need 200 individuals/genotype to have sufficient power to avoid a type 2 error when trying to detect a difference of 3–5%.

### Linking more detailed body composition measures to metabolic rates

Konarzewski and Diamond (1995) compared the BMR and masses of the heart, kidneys, liver, and small intestines in six strains of mice and found that although these four organs accounted for only 17% on average of the tissue mass of the mouse they accounted for 52% of the variation in BMR. These data suggest that effects of genotype on metabolism may be mediated by effects on sizes of key organs rather than on metabolism *per se*. Using ANCOVA with the body partitioned into lean and fat mass would not be able to eliminate this as a possible explanation for a significant genotype effect on energy expenditure.

To separate these organ size effects from an effect on tissue level metabolic rates it would be necessary to measure the animals and then sacrifice them to remove and weigh their organs. This has been done on relatively few occasions in mice. Two examples are Speakman and Johnson (2000) and Selman et al. (2001). In the first study we aimed to explore the links between organ size variation and variability in the metabolism of lactating mice. In the second we aimed to investigate the contribution of organ size differences to the different metabolic rates of mice that had been selected for 38 generations for high and low food intake normalized for body mass (Hastings et al., 1997). McDevitt and Speakman (1994) also used this approach to explore the basis of cold acclimation changes in BMR in voles.

There are two separate analytical approaches that can be taken with these types of data. The first is to simply extend the multiple linear regression model to include more predictors – replacing the fat and lean tissue masses with the masses of the individual dissected organs. Selman et al. (2001) used this approach and found that in addition to the empty carcass the metabolism of the high and low food intake strains was significantly positively related to four organs (the tail, liver, spleen, and heart) the dominant effect being of the liver. In fact the strain differences in resting metabolism could be completely accounted for the by the strain differences in liver size. Given that the two strains had been selected for high and low food intake differences in the size of the liver between the strains, which were then linked to the differences in metabolic rate between the stains was not surprising. However, this study illustrates that even when there are large strain (or genotype) effects on metabolic rate these do not necessarily reflect tissue level metabolic rates but may be explained by relative differences in organ sizes. As far as I am aware nobody has yet eliminated such a possible explanation for any genotype effect on metabolic rate by measuring the sizes of all the organs in the respective genotyped animals following indirect calorimetric measurements.

**Figure 14:**
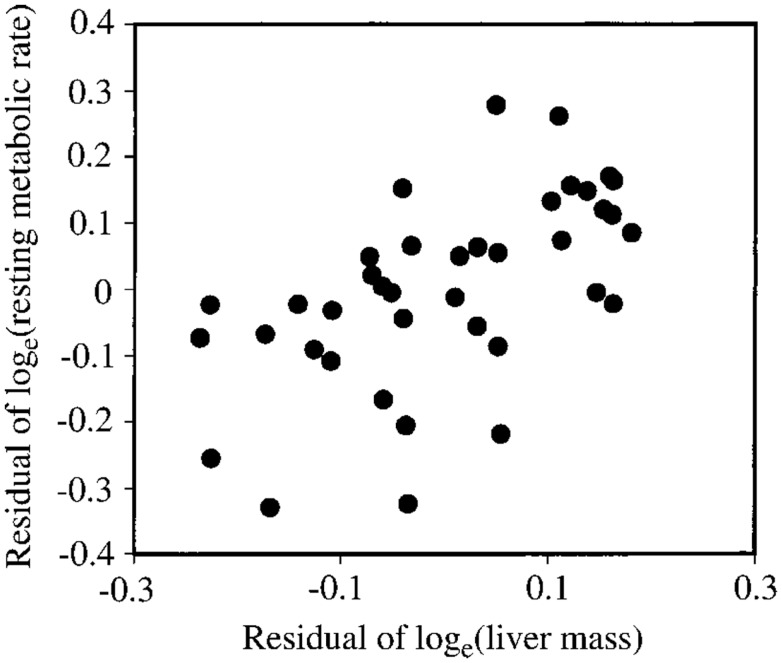
**Residual metabolic rate plotted against residual liver mass (both with the covariable effects of total body mass removed) across 39 individual mice from two strains (from Selman et al., 2001)**.

There are two major issues however with this approach. The first is the ratio of variables to observations (Even and Nadkarni, 2012). Mice can be dissected into a large number of distinct organs. Selman et al. (2001), for example, split their mice into 19 different components. However, they only measured 39 individual mice hence the ratio of measurements to variables was just over 2. Ideally in this type of multiple regression model the target to aim for is a ratio of above 6. The second problem is statistical inference in multiple linear regression models is only possible if the predictor variables are independent of each other. Yet organ masses are clearly correlated. Bigger individuals have the tendency for all their organs to be on the large side, and smaller animals show the opposite trend. To overcome this problem one method is to express the relationship between each organ size and the total body mass and then calculate the residual values to the fitted regression. One can also do the same for the RMR measurement and then include the residual masses of the organs into a multiple regression model as predictors and the residual RMR measurement as the dependent variable. When Selman et al. (2001) did this the effect of the liver remained highly significant (Figure 14), but the effects of the tail, spleen, and heart were no longer significant suggesting their effects in the previous analysis were artifacts of being correlated to the total body weight. Interestingly, however, a negative effect of pelage weight emerged in this analysis, which was not found in the original analysis.

To overcome the first problem of the ratio of measurements to variables there are two different approaches that can be employed. The first is to group different organs and tissues together in functional groups to reduce the number of variables. Selman et al. (2001) for example reduced their 19 tissues to five functional groups (making the ratio of variables to measurements about eight) and repeated the analysis. The liver again emerged as the only significant predictor. A different approach was used by Speakman and Johnson (2000) in 59 lactating mice that were dissected into 18 different organs and tissues. In this group a principal components analysis was run to compress the 18 variables into five principal components which still retained 80% of the original variance. The only scores to enter the stepwise regression with RMRt as the dependent variable were those for PC1, which was a general body size component. When residual RMRt was used even these scores were not significant.

## Summary

Measuring the energy metabolism of mice is a key skill that is necessary to understand the impact of genetic manipulations, or of drug and compound treatments, on energy metabolism. Generally daily energy expenditure can be partitioned into different components: basal or resting metabolism, the costs of thermoregulation and physical activity, and the thermic effect of feeding. Measuring daily energy demands is best performed using large chambers that permit the animal to replicate its home cage behavior. These larger chambers may be linked to a single analyzer or have several chambers sequentially monitored by one analyzer. Decomposing the outputs from such systems to yield the component metabolic rates is currently not feasible with the required degree of accuracy. Component metabolic rates are rather better determined using small fast washout chambers where a single chamber is linked to a single analyzer. Outputs from both types of system pose challenges for analysis, in particular how best to correct for differences in body mass and composition between individuals. Generally the most appropriate statistical approach for treatment of such data is ANCOVA.

## Conflict of Interest Statement

The authors declare that the research was conducted in the absence of any commercial or financial relationships that could be construed as a potential conflict of interest.
